# Conformational Dynamics in the Cell Membrane Interactions of Bispecific Targeted Degrader Therapeutics

**DOI:** 10.1021/acs.jmedchem.5c01499

**Published:** 2025-12-10

**Authors:** Emma Inganäs, Hanna Lavén, Rémi Caraballo, Fredrik Klingegård, MyLan Eklund, Linda Andersson, Constanze Hilgendorf, Pär Matsson

**Affiliations:** † Unit for Pharmacokinetics and Drug Metabolism, Department of Pharmacology, Sahlgrenska Academy, 70712 University of Gothenburg, 405 30 Gothenburg, Sweden; ‡ SciLifeLab Drug Discovery and Development, Medicinal Chemistry Hit2Lead, 463758 Karolinska Institute, 171 65 Solna, Sweden; § DMPK, Early Research and Development, Cardiovascular, Renal and Metabolism, BioPharmaceuticals R&D, AstraZeneca, 431 53 Gothenburg, Sweden; ∥ SciLifeLab Gothenburg, University of Gothenburg, 405 30 Gothenburg, Sweden

## Abstract

Proteolysis targeting chimeras (PROTACs) offer vast new therapeutic opportunities, but their physicochemical properties are difficult to combine with optimal cell permeability and exposure at the target sites. We have systematically analyzed a data set of more than 3500 PROTACs to investigate how the choice of ubiquitin E3 ligands, linker design, and global molecular properties can be optimized to achieve the desired cell membrane interactions and ultimately membrane permeability and intracellular exposure. We find that conformational flexibility leads to environment-dependent shielding of polar functions and improved interactions with cell membranes but that at the same time, extended, linear conformations within the membrane are beneficial. Linker composition was a major factor in determining the folding propensity. Collectively, our results suggest that strategies to rationally design linkers and to shield polarity selectively within the protein-of-interest (POI) ligand and/or E3 ligand domains rather than more extensive folding may be beneficial in the design of permeable and effective PROTACs.

## Graphical abstract

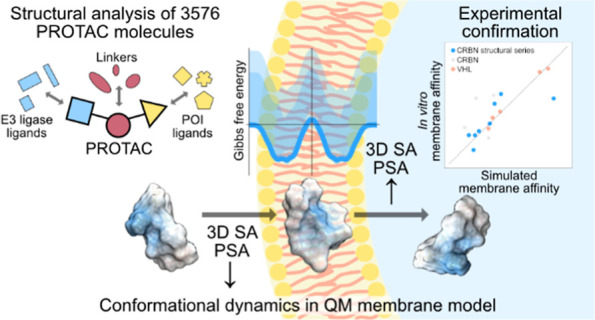

## Introduction

Targeted protein degradation has become a valuable new tool in the drug discovery and chemical biology toolbox. Targeted degraders, or proteolysis targeting chimeras (PROTACs), work by hijacking the cell’s ubiquitin–proteasomal degradation system: bispecific ligands simultaneously bind the target protein and an E3 ligase, and the induced proximity leads to selective ubiquitination and subsequent degradation of the target.^1–8^

PROTACs have unique characteristics that set them apart from traditional therapeutic modalities.^9^ Most importantly, they circumvent the need for constant target occupancy: conventional drugs are only effective when all or most target molecules are occupied and when these are bound in a way that alters the function of the target protein. In contrast, a single PROTAC molecule can iteratively bind to and induce degradation of multiple copies of the target protein in a substoichiometric, catalytic fashion.^2,10^

As with all drugs that target intracellular biomolecules, PROTACs must be able to cross the plasma membrane to elicit the therapeutic response. Importantly, not all of the drugs that enter the cell interior are available to bind to intracellular targets. Typically, a variable and unpredictable fraction of the drug is bound to cellular components or trapped in cellular organelles.^11–13^ We have previously shown that free intracellular drug concentrations are an excellent predictor of intracellular drug potency for various intracellular targets^11–13^ and that the pharmacologically active free intracellular drug fraction is critically determined by nonspecific binding to (sub)­cellular phospholipid membranes.^11,14,15^ Recent investigations revealed widespread and extensive tissue distribution in rodents for two PROTACs, one CRBN-directed^16^ (ARV-110) and one VHL-directed^17^ (A947), with tissue-to-plasma distribution ratios (Kp) indicating substantial binding to tissue components. Indeed, several clinical trials of PROTACs have either explicitly reported high distribution volumes^18^ or long half-lives (>24 h),^18–21^ which are indicative of extensive distribution and/or low plasma clearance.

The bispecific affinity of the degrader molecule leads to a considerably greater molecular size than in traditional, cell permeable molecules, significantly restricting intracellular exposure.^22^ Furthermore, the larger size of PROTACs is typically accompanied by corresponding increases in additional physicochemical descriptors, such as lipophilicity and the number of hydrogen bonding functionalities,^22^ often considerably overstepping the boundaries of traditional, rule-of-5 compliant drug-like chemical space. Thus, PROTACs are situated in a challenging region of chemical space, where large size in combination with polar functionalities may limit cell permeability, whereas more lipophilic PROTACs can exhibit both problematic solubility and excessive binding to cellular and subcellular lipid membranes, potentially affecting their pharmacokinetics and pharmacological activity.^23^ Additionally and importantly, these same physicochemical properties also lead to extensive binding to assay plastics, often severely complicating measurement using conventional experimental setups.^24,25^

We and others previously demonstrated that cell permeability in macrocyclic molecules^26,27^ and in orally available beyond-rule-of-5 drugs^28,29^ is improved by, and sometimes contingent on, the ability to adaptively expose or shield molecular features depending on the surrounding environment. Such environment-dependent conformational sampling, referred to as molecular chameleonicity, has since been demonstrated in specific case studies also for PROTACs, indicating that similar rules govern their cell permeability compared to those for other beyond-rule-of-5 molecules.^30–32^

Numerous studies have demonstrated that the length and type of the linker connecting the two protein ligands can have dramatic effects on degradation efficiency.^1,33,34^ In addition, the choice of linker chemistry and length has been shown to lead to large differences in permeability in structurally similar PROTAC series^35,36^ and that permeability differences may be associated with conformational flexibility in the linker region.^30,31^ However, whether these initial findings generalize to other PROTACs and can be used in prospective drug design remains unknown.

We have, therefore, collected and analyzed a considerable proportion of the PROTACs available to date in the public domain, with the aim to define structural features and molecular properties that govern their interactions with and transport across cellular membranes.

## Results and Discussion

### Physicochemical Characteristics of PROTACs

A database comprising 3576 PROTAC molecules was collated from public data sources and complemented with the recent PROTACs literature.^22,37^ Of these, 2304 (64%) contained a cereblon-binding domain (CRBN), 1195 (33%) contained a Von Hippel–Lindau binding domain (VHL) (Table 1), and the rest (3%) were reported to bind to other E3 ligases (Table S1). Additionally, 2.4% of the VHL-binding PROTACs had CRBN as the degradation target and, thus, also contained a CRBN-binding POI ligand.

**Table 1:** Molecular Properties of Literature PROTACs (*n* = 3499)^a^

CRBN-binding PROTACs				
	Full molecule	E3 ligand	Linker	POI ligand
n types	2304	60	1174	444
MW^b^ [Da]	874 (409–2009)	272 (188–511)	182 (0–647)	423 (60–1581)
*c* Log *P* ^c^	4.4 (−7.9–12.3)	0 (−1.3–3.4)	0.7 (−1.7–7)	3.8 (−6.9–8.6)
TPSA^d^ [Å2]	214 (87–793)	96 (46–175)	37 (0–150)	82 (3–654)
HBD^e^	4 (1–25)	2 (0–2)	1 (0–3)	2 (0–23)
HBA^f^	13 (4–30)	5 (2–11)	3 (0–12)	6 (1–24)
nRotB^g^	17 (4–62)	2 (1–8)	8 (0–38)	5 (0–52)

VHL-binding PROTACs				
	Full molecule	E3 ligand	Linker	POI ligand
n types	1195	74	543	256
MW^b^ [Da]	1051 (717–1684)	458 (390–705)	162 (0–777)	418 (167–989)
cLogP^c^	7.1 (0.9–14.4)	2.4 (0.4–4.6)	1 (−1.3–5.6)	4 (−2.4–9)
TPSA^d^ [Å2]	217 (124–356)	112 (91–152)	24 (0–149)	80 (12–244)
HBD^e^	5 (2–10)	3 (2–4)	0 (0–2)	2 (0–7)
HBA^f^	14 (8–25)	6 (4–11)	2 (0–14)	6 (1–12)
nRotB^g^	21 (5–44)	6 (5–12)	8 (0–32)	5 (0–18)

a Summary of molecular descriptors (median (min–max range)) of full PROTAC molecules and for the separated E3 ligand, linker, and POI ligand domains for CRBN-binding and VHL-binding PROTACs.

b Molecular weight.

c Calculated Log *P*.

d Topological surface area.

e Hydrogen bond donors.

f Hydrogen bond acceptors.

g Number of rotatable bonds.

VHL-binding PROTACs were, on average, larger and more lipophilic (as assessed by their molecular weight, MW, and calculated octanol–water partition coefficients, *c* Log *P*; Table 1). The number of hydrogen bond acceptors and donors (HBA/HBD) were similar in VHL- and CRBN-binding PROTACs, and thus, it follows that their topological polar surface areas (TPSA) were approximately equal. Consequently, the increased size results mostly from nonpolar atoms, explaining the substantially greater lipophilicity in VHL-binding PROTACs (Table 1).

By separating each of the compiled PROTAC structures into their three functional parts, we could assess the relative contributions of each to the properties of the whole molecule. Excluding stereochemical variations, a total of 127 different E3 ligands, 1540 different linkers, and 591 different POI ligands were identified in the data set. While POI ligands and linkers were similar in size in all classes of PROTACs, the greater overall size of VHL-directed PROTACs than of CRBN-binding PROTACs originated primarily from their substantially larger E3 ligands.

On average, the POI ligand was the most lipophilic part of the PROTAC, and VHL-binding domains were more lipophilic than CRBN-binding domains. The VHL-binding domains also contained more rotatable bonds, indicating greater conformational flexibility than that in CRBN ligands. Notably, HBA, HBD, and TPSA were similar for the different E3 ligands, despite their considerable size difference. These polarity descriptors were also similar for the POI ligand domains in VHL- and CRBN-binding PROTACs.

### Conformation-Dependent Polarity Shielding

We previously demonstrated that large and conformationally flexible (“beyond-rule-of-5”) molecules dynamically expose or shield polar atoms depending on the surrounding environment (“molecular chameleonicity”).^26,29^ This has since also been demonstrated in smaller case studies of PROTACs.^30,31,38,39^ To assess if such conformation-dependent polarity is a recurrent feature in PROTACs and how it in turn affects membrane interactions, we systematically analyzed more than 900 chemically diverse PROTACs representative of the full collection, selected to cover the PROTAC chemical space, as described by a principal component analysis (Figure S1A). The subset was subjected to extensive molecular mechanics conformational sampling in water and chloroform environments to explore the structural dynamics of PROTACs in relevant biological compartments.

Fewer distinct low-energy conformations were identified in chloroform than in water for the vast majority of all PROTACs, indicating a restricted conformational sampling in the nonpolar, membrane-mimicking solvent (Table S2). Since these were implicit-solvent calculations, the different conformational diversity reflects constraints arising from the electronegativity of the respective solvents rather than steric effects and lateral pressure that would also contribute to a physical lipid membrane environment. Our results were consistent with NMR-derived conformations for a PROTAC molecule reported by Atilaw et al.,^30^ for which a limited number of conformations were observed in each of the three studied solvents (chloroform, DMSO, and DMSO–D_2_O), with greater conformational variations in the polar solvents and with little overlap between the respective conformation ensembles.

In general, the conformations exposed greater solvent-accessible PSA in water than in chloroform, irrespective of which E3 ligand domain the PROTAC contained. Further, CRBN-binding PROTACs exposed more polar surface in either solvent than did VHL-binding ones (Table S2), despite containing similar numbers of polar atoms and having similar topological PSA. It thus appears that VHL-binding PROTACs are able to hide more polar surface area than CRBN-binding ones.

To further understand the degree of conformation-dependent polarity shielding in PROTACs, the difference between the maximum and minimum PSA among each molecule’s conformations was calculated and normalized to the maximum PSA (to account for between-compound differences in the “shieldable surface area” due to their different polar atom contents). The within-compound ranges of the solvent-exposed PSA varied between 2% and 80% of the maximum exposed PSA for CRBN-binding PROTACs, and up to 300 Å^2^ (6–300 Å^2^) PSA was found to be hidden in the most shielded conformations. For VHL-binding PROTACs, these numbers were instead between 13% and 76%, with up to 248 Å^2^ (25–248 Å^2^) PSA shielded. Thus, we conclude that polarity shielding is a common feature of PROTACs regardless of which E3 ligase is targeted but that the ability to shield polar groups varies greatly (Figure 1A–D).

**Figure 1:**
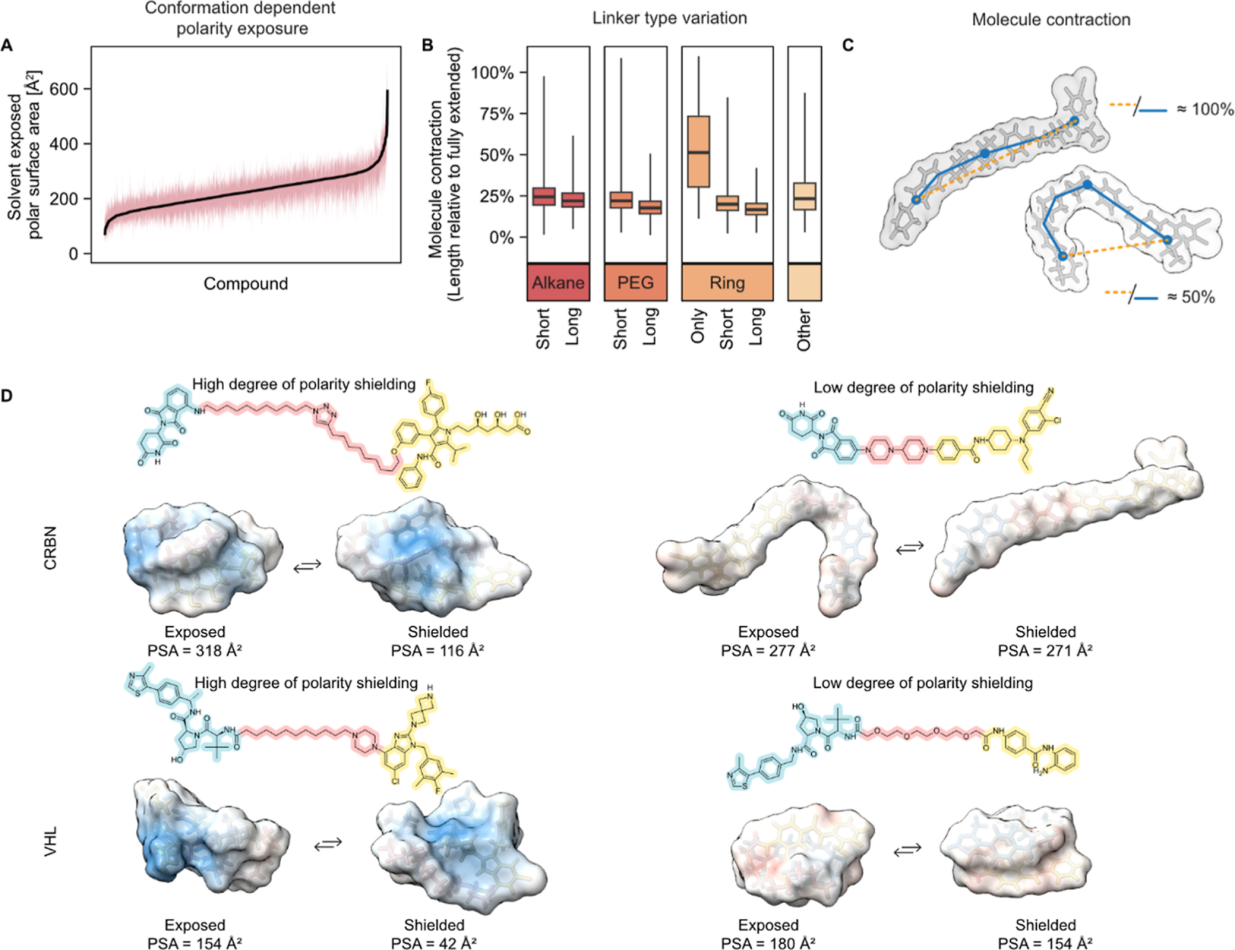
Environment-dependent exposure of polar atoms and molecule contraction. (A) Large variability is seen in the ability of different PROTACs to shield polar surface area. The black solid line indicates the median, and the red shaded area indicates the min–max range (*n* = 911). (B) Differences in molecule contraction among the different linker type groups (*n* = 911 PROTACs). The black solid line indicates the median, and the bottom and upper box limits are the 25th and 75th percentile, respectively. Whiskers indicate the minimum and maximum values. (C) Explanation of the molecule contraction parameter. (D) Examples of CRBN-binding (top panels) and VHL-binding (bottom panels) molecules with high degree (left panels) and low degree (right panels) of shielded polar surface area.

### Linker Characteristics and Conformational Flexibility

While the POI ligand and E3 ligand domains have clear and direct roles in the proximity-inducing effect, it is becoming clear that the linker region can also markedly affect degradation efficiency by steering the distance and relative orientation in the ternary complex.^1,33,34^ Much less is known about how linker chemistry and structure affect membrane interactions and cellular exposure.

To enable delineation of such an impact, the separated linker structures were further analyzed to identify commonly occurring substructures. Visual inspection yielded a list of motives, including saturated alkyl and polyethylene glycol (PEG) chains of increasing lengths, discrete substructures such as azo-, alkyne-, and sulfur-containing moieties, and various ring systems, which were subsequently mapped onto each of the identified linkers using a custom RDKit script. The most common linker substructures were alkyl chains (found in approximately 37% of all linkers) and PEG chains (found in one-third of all linkers) (Figure S2A,B). Frequencies of both linker motifs decreased with an increase in motif length. Further, 47% of the linkers contained at least one amide bond, and 56% contained some kind of ring system, the most common of which were piperazines and triazoles (Figure S2A). Notably, the predominance of simple aliphatic and PEG-based linkers in reported PROTACs likely results from a bias in the literature toward early-stage discovery projects primarily aimed at identifying suitable POI ligands and connection vectors, whereas reported clinical-stage PROTACs often contain shorter, more rigid ring-containing linkers. In our collated literature data set, such linkers were less frequent and observed in 6% of the molecules.

Conformational flexibility in PROTACs can arise from rearrangements in any combination of the three constituent parts—the E3 ligand, linker, and POI ligand domain—as well as in their movement relative to each other. To assess the underlying dynamics leading to variable conformational sampling in different PROTACs, we calculated two complementary parameters: “linker contraction”, which describes the fractional reduction in linker length compared to its fully extended form, and “molecule contraction”, which describes how the entire structure folds by comparing the distance between the centers-of-mass for the E3 ligand and POI ligand domains in relation to the fully extended structure (Figures 1C and S3).

We characterized the ability of each of the different linker classes to form folded structures and found that PEG-based linkers of length ≥3 monomers (i.e., ≥9 atom chains), with or without ring systems, gave the most folded molecules (Figure 1B). This was consistent with NMR-derived conformations for two PEG-linked PROTACs, where longer chains were shown to favor the gauche effect and a more folded structure.^31^ In contrast, linkers consisting solely of rings yielded conformations that, on average, were close to being fully stretched (Figure 1B). Alkyl-based linkers and ring systems connected through short linear chains resulted in modest folding.

We found that reduction in exposed PSA correlated more strongly with an overall folding of the molecules (*R* ^2^ = 0.02–0.25, Figure S2C) than with contraction of the linker region (*R* ^2^ = 0–0.05, Figure S2D), indicating that polarity shielding results from conformational changes that extend outside the linker itself and that also involve rearrangement in the POI ligand and E3 ligand domains. Thus, the impact of changing linker chemistry on the overall molecule properties will likely depend strongly on the chemical nature of the E3 ligand and POI ligand domains.

### Interactions with and Permeation across Model Cell Membranes

Permeation across cell membranes is essential for PROTACs to reach their sites-of-action, and importantly, binding to subcellular membranes also governs how much of the internalized PROTAC is freely diffusing in the cytosol and available to form the active ternary complex and how much is sequestered and pharmacologically inaccessible. Consequently, binding to membranes is a dominating factor influencing the overall distribution of drugs to peripheral tissues and a likely contributor to the large volumes of distribution reported for clinical-stage PROTACs.^18–21^

To examine the relationship between PROTAC chemistry and their interactions with cell membranes more closely, we sourced a chemically diverse, representative subset (Figure 2A) from commercial vendors and measured their binding to HEK293 cell homogenates using equilibrium dialysis using methods previously introduced by us^11,12^ and widely adopted in the field.^40–43^ Matched molecular pairs were included, in which POI and E3 ligands were kept constant and the length or chemistry of the linker varied. All sourced compounds are reported to degrade their respective POIs, ensuring the exploration of functionally relevant PROTAC chemistries.^7,8,44–50^ Longer alkane linkers resulted in a higher degree of binding (4.6-fold higher bound-to-unbound concentration ratios for compound **8** than for **9**), and a PEG-based linker resulted in lower membrane binding than an alkane-based one of comparable length (5.3-fold lower for compound **10** than for **7**). Thus, it is clear that the choice of linker can substantially influence interactions with cellular membranes.

**Figure 2:**
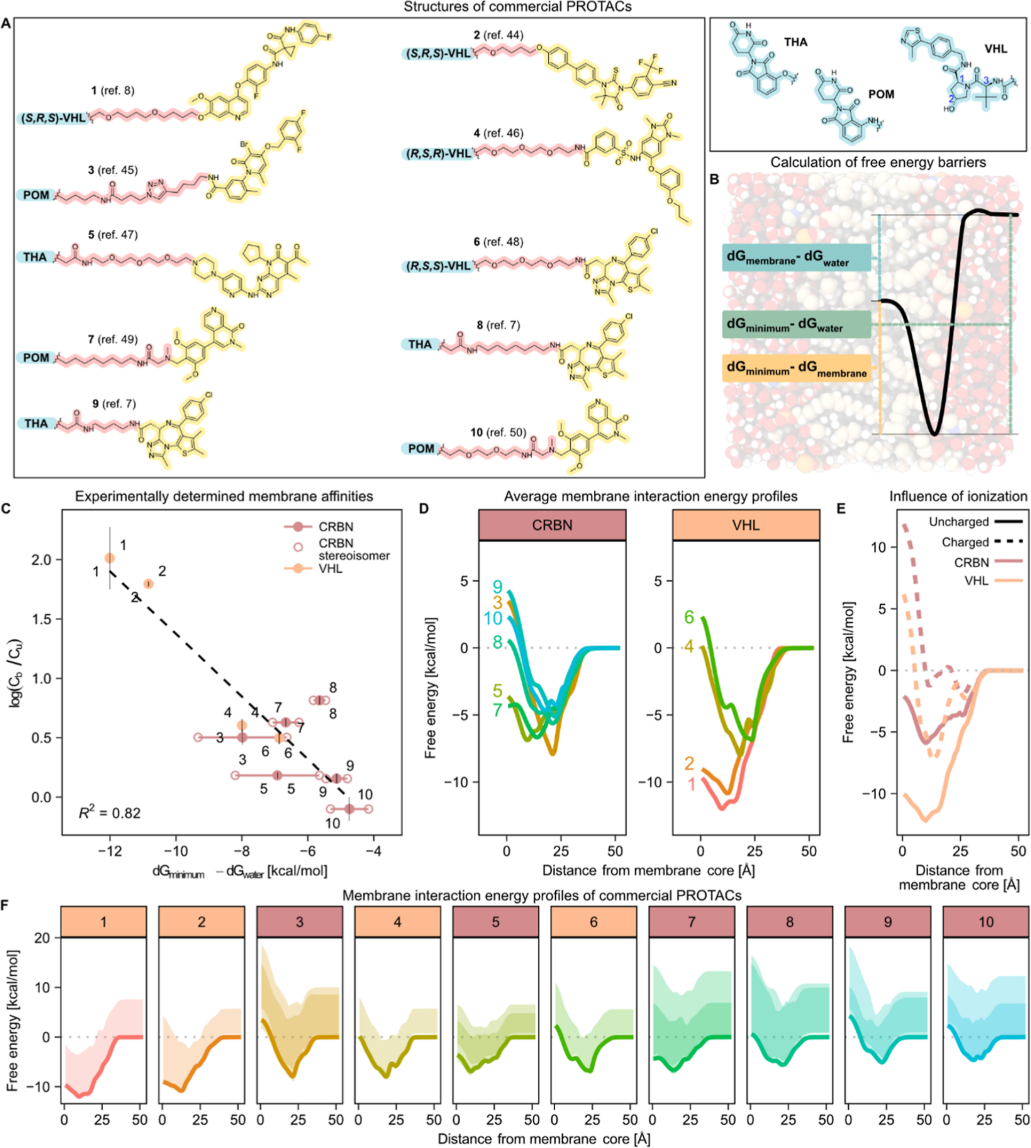
Interactions of PROTACs with in vitro and simulated cell membranes. (A) PROTAC molecular structures.^7,8,44–50^ (B) Schematic illustration of a typical free energy profile in a simulated DMPC membrane with calculated energy barriers. (C) The depth of energy minima correlates well with experimental binding to the HEK293 cell homogenate, expressed as the logarithm of bound-to-unbound concentration ratios, log­(*C*_b_/*C*_u_) (mean ± SD, *n* = 3). Full circles show the average, and open circles show the individual stereoisomers. (D) Average free energy profiles for CRBN-binding PROTACs (left panel) and VHL-binding PROTACs (right panel). (E) Free energy profiles of example PROTACs (compounds **1** and **5**) in their uncharged (solid lines) and charged states (dashed lines). (F) Free energy profiles of individual PROTACs with conformational variability. Solid lines indicate the Boltzmann-weighted energy profiles, averaged across molecule stereoisomers. Shaded areas show the min–max range of free energies across all conformations for each individual stereoisomer. Darker shades indicate energy regions covered by both stereoisomers.

The test compounds were further analyzed in silico with the aim of quantitating the energetics of membrane interactions and identifying the main barriers (Figure 2B) and preferential orientations and conformations adopted in the membrane binding. We used the continuum solvation model (COSMO), in which density functional theory (DFT)-based calculations are applied to derive surface polarization charge densities for each type of molecule in a multicomponent mixture, and their miscibility was subsequently assessed through statistical thermodynamics.^51^ COSMO methodology has previously been extensively benchmarked with both smaller and larger molecule solutes and accurately recapitulates both experimentally measured solute-membrane affinities and permeation barriers^52–54^ and computationally intensive molecular dynamics simulation protocols.^53,55,56^ To assess conformation-dependent effects, charge densities were calculated for representative low-energy conformations of each analyzed PROTAC obtained from molecular mechanics calculations, and a Boltzmann weighting approach was used to derive free energy profiles for the interaction of each compound’s conformational ensemble with a DMPC (1,2-dimyristoyl-sn-glycero-3-phosphocholine) phospholipid membrane (Figure 2D,F).

We found that deeper energy minima strongly correlated with increased binding to the cell homogenate (Figure 2C), in line with similar calculations for small organic solutes where COSMO-derived energy minima have been shown to reflect affinity to model phospholipid membranes.^53^ VHL-binding PROTACs were, on average, more bound to cell homogenates and had correspondingly more pronounced energy minima than CRBN-binding ones.

As expected, ionized species showed extensive energy barriers at the membrane core compared to the un-ionized species of the same PROTACs (exemplified in Figure 2E), resulting in extremely low probabilities (<0.001 ppm; Boltzmann distribution) of finding the ionized species at the center of the membrane bilayer. Subsequent analyses of membrane interaction propensity were therefore focused solely on un-ionized species.

We also measured the passive transepithelial permeability of the 10 compounds in a transporter-inhibited Caco-2 assay setup. The compounds spanned approximately 3 orders of magnitude in permeability (<0.055–65 × 10^–6^ cm/s). Dividing the PROTACs into groups of high, medium, and low permeability revealed a correlation with the calculated membrane energy profiles (Figure 3A,B). High-permeability PROTACs were characterized by comparatively shallow energy minima at the membrane–water interface and low barriers at the membrane center. In the moderate-permeability group, the energy minima were lower and the membrane–center barrier was more varied. The low-permeability PROTACs separated into two distinct patterns, either presenting a very low energy minimum or a substantially higher energy at the membrane center than in water. Both patterns thus represent high energy barriers to leaving the membrane–water interface. Overall, the passive cell permeability was well described by the dominant energy barrier for each compound (Figure 3C). Our results align with previous reports for small-molecule membrane permeation^57^ and demonstrate that PROTAC permeability can be limited either by a high barrier to transfer between bilayer leaflets (“flip-flop”) or by a deep energy minimum at the membrane interface, restricting the release of permeating molecules to water.

**Figure 3:**
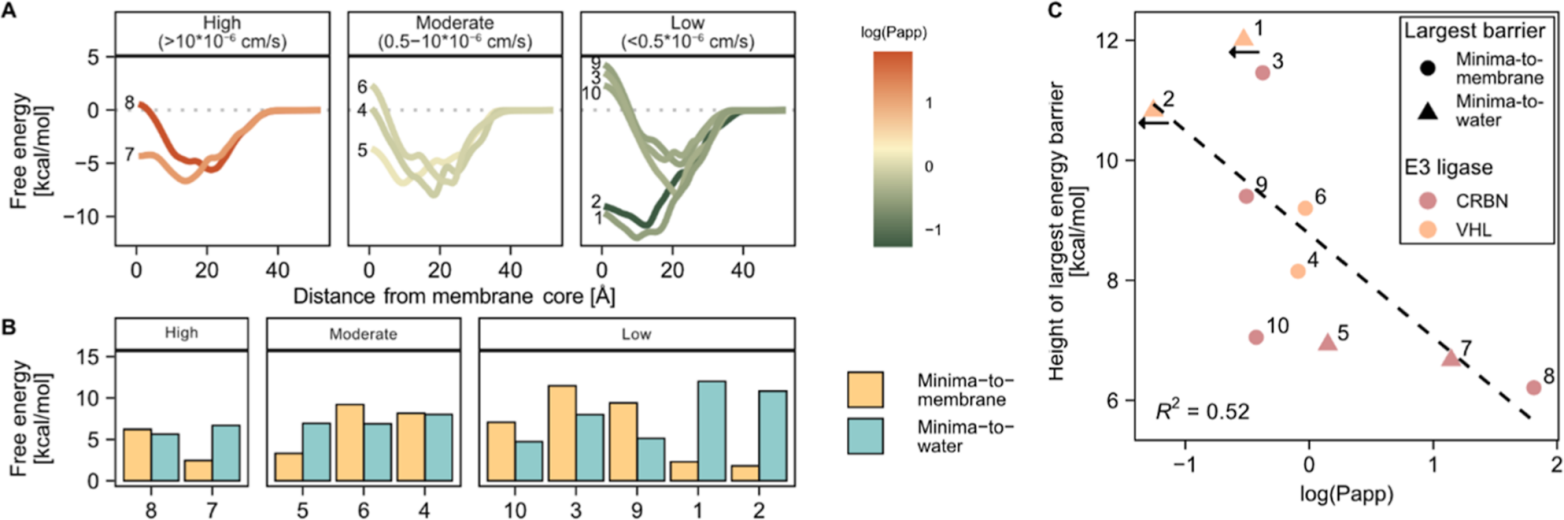
Passive Caco-2 cell monolayer permeabilities. (A) Free energy profiles of PROTACs grouped based on passive cell permeability (high, moderate, or low). The profiles are colored according to permeability. (B) Energy barriers to leaving the energy minimum at the membrane–water interface. The barrier from the energy minima to the membrane core (yellow) or to water (blue) is indicated for each compound. Compounds are ordered by their passive permeability, high to low from the left. (C) Correlation between the height of the largest energy barrier and passive cell permeability. Circles indicate compounds for which the minima-to-membrane barrier is the largest, and triangles indicate compounds for which the minima-to-water barrier is the largest. Colors indicate the targeted E3 ligase (CRBN; red, VHL; orange). Left-pointing arrows indicate compounds with receiver-side concentrations near the limit of quantitation and for which the actual permeability may be lower than indicated.

### Membrane Interactions of Structurally Diverse PROTACs

Encouraged by the correlations between experimentally measured Caco-2 cell permeability and affinities for isolated cell membranes to COSMO-derived free energy profiles, we extended our analysis to a more comprehensive set of 210 representative molecules to probe in detail what structural and chemical features govern the respective profile shapes and energy barriers. This representative subset was selected from a principal component analysis to cover the physicochemical space of the full PROTAC database and also included structural series in which each of the three PROTAC structural components was varied in isolation (Figure S1B).

Increased molecule lipophilicity (assessed with the two-dimensional *c* Log *P* estimate) led, on average, to deeper energy minima at the water–lipid interface and lower barriers for traversing the membrane core (Figure 4A). Weaker, positive correlations were seen between energy barrier height and the polar surface area exposed in the most shielded conformation, indicating that the ability to form low-polarity conformations is beneficial for interactions in the membrane core (Figure 4B). No clear correlations were seen for the other fundamental molecular properties assessed (MW, HBD, HBA, TPSA, and nRotB). This contrasts with observations for oral absorption in rodents, which was substantially reduced in PROTACs with more than three HBDs or high MW (>950 Da).^58^ Notably, similar observations in beyond-rule-of-5 macrocycles were explained by extensive transporter-mediated efflux at higher HBD counts rather than by a reduced passive membrane permeability,^26^ and a corresponding influence from efflux transporters on PROTAC intestinal permeability and absorption is likely given their physicochemical characteristics.^59,60^

**Figure 4:**
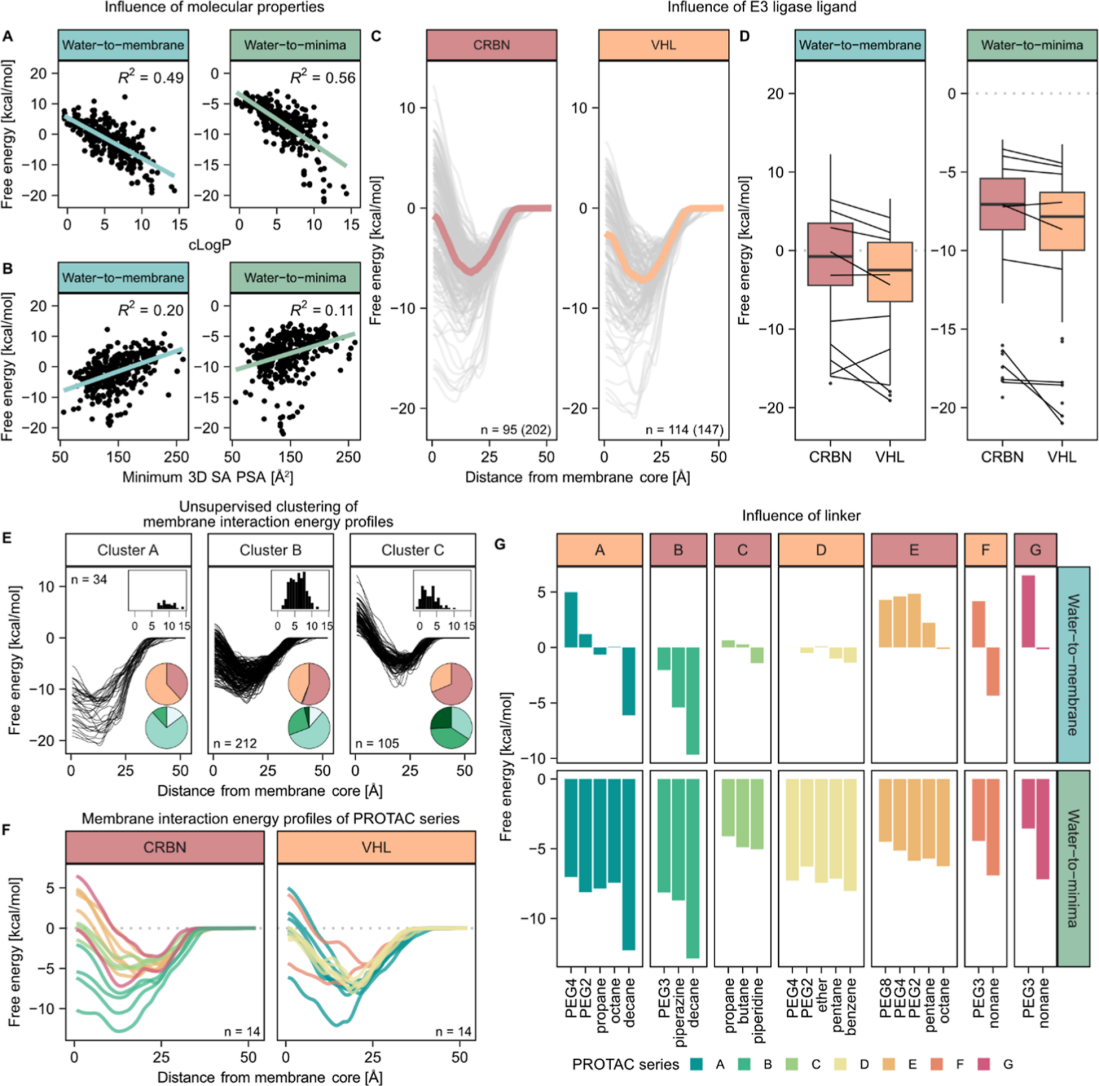
Interactions of representative PROTACs (*n* = 210 discrete molecules; 351 stereoisomers) with simulated phospholipid membranes. (A) High *c* Log *P* correlates with lower energy minima and barrier in the membrane core. (B) Lower minimum solvent-accessible polar surface area correlates moderately with lower energy in the membrane core. (C) Free energy profiles of CRBN- and VHL-binding PROTACs. Median profile in red/orange, generated from the median energy at each depth in the membrane. Per-compound profiles are shown in gray. Numbers indicate the count of discrete molecules analyzed, with stereoisomer counts in parentheses. (D) Free energy barriers of CRBN- and VHL-binding PROTACs at the membrane core and minima. The black solid line indicates the median, and the bottom and upper box limits are the 25th and 75th percentile, respectively. Whiskers extend to 1.5 multiples of the interquartile length. Lines between the CRBN and VHL boxes connect structurally analogous compounds that differ only in the E3 ligand. The molecular structures of the matched pairs are shown in Figure S7. Energy barrier explanation is given in figure legend 2B. (E) Unsupervised clustering of free energy profiles, *c* Log *P* distributions are indicated in histogram inserts. Top pie charts show the proportion of VHL-binding (orange), CRBN-binding (red), and IAP-binding (gray) PROTACs. Bottom pie charts show minimum 3D SA PSA; <100 Å^2^ (white), 100–150 Å^2^ (light green), 150–200 Å^2^ (green) or >200 Å^2^ (dark green). Numbers indicate the count of stereoisomers in each cluster. (F) Free energy profiles, averaged across stereoisomers, of structurally similar series of PROTACs that differ only in the linker. Each series is represented by its own color, as indicated in the legend in G. Numbers indicate the count of discrete molecules. (G) Influence of the linker motif in different PROTAC series (color on the label indicates if the molecule is CRBN- (red) or VHL-binding (orange)). Compounds are ordered left to right by their TPSA (high to low), and those with the same TPSA are internally ranked by their lipophilicity (low to high). The molecular structures of series A–G can be found in Figure S6.

Consistent with the experimental data set (**1**–**10**), energy minima at the interface were generally lower for VHL- than for CRBN-directed PROTACs, and the energy barrier at the membrane core was also lower for VHL-directed PROTACs. Analysis of 10 matched molecule pairs, which within each pair share identical POI ligands and linkers but vary in the E3 ligase ligand, confirms the global findings that VHL-PROTACs tend to have lower energy barriers than their CRBN counterparts (Figure 4C,D). Correspondingly, keeping the linker and ligase ligand constant, deeper energy minima and lower energy barriers at the membrane core were observed with the more lipophilic POI ligand (Figure S4). This was true for both VHL-and CRBN-directed PROTACs.

Using unsupervised clustering to identify groups of PROTACs that share similar energy profiles further confirmed the impact of molecule lipophilicity (Figure 4E). Furthermore, the proportion of CRBN-binding PROTACs was greater in the cluster with a more pronounced membrane penetration barrier, and PROTACs with lower solvent-accessible polar surface area were more common in the cluster with a small penetration barrier.

Linker length and chemistry had less clear-cut effect on the PROTAC–membrane interaction energies. Longer PEG-based linkers were more common in cluster C with greater penetration barriers, whereas rigid and lipophilic linkers (ring- and alkyl-based linkers) were more common in the low-barrier cluster A (Figure S5). Focused analyses of the series with identical POI and E3 ligands but variations in the linkers (Figure S6) showed that long PEG-based linkers gave greater penetration barriers, while long alkanes yielded, on average, lower energy minima than other classes, in line with the results from the global clustering analysis (Figure 4F,G).

### Orientation and Polarity Exposure in PROTAC–Membrane Interactions

The observed environment-dependent exposure of polar atoms (Figure 1A,D) indicated functionally selective conformational sampling as the PROTACs partition into the hydrophobic environment of the membrane core, but the implicit-solvent calculations used are not sufficient to reveal how the permeating molecules orient themselves as they enter the membrane. We therefore extended our analysis, examining the preferential orientation of PROTACs as a function of penetration depth in the heterogeneous QM-derived membrane model (Figure 5A–E).

**Figure 5:**
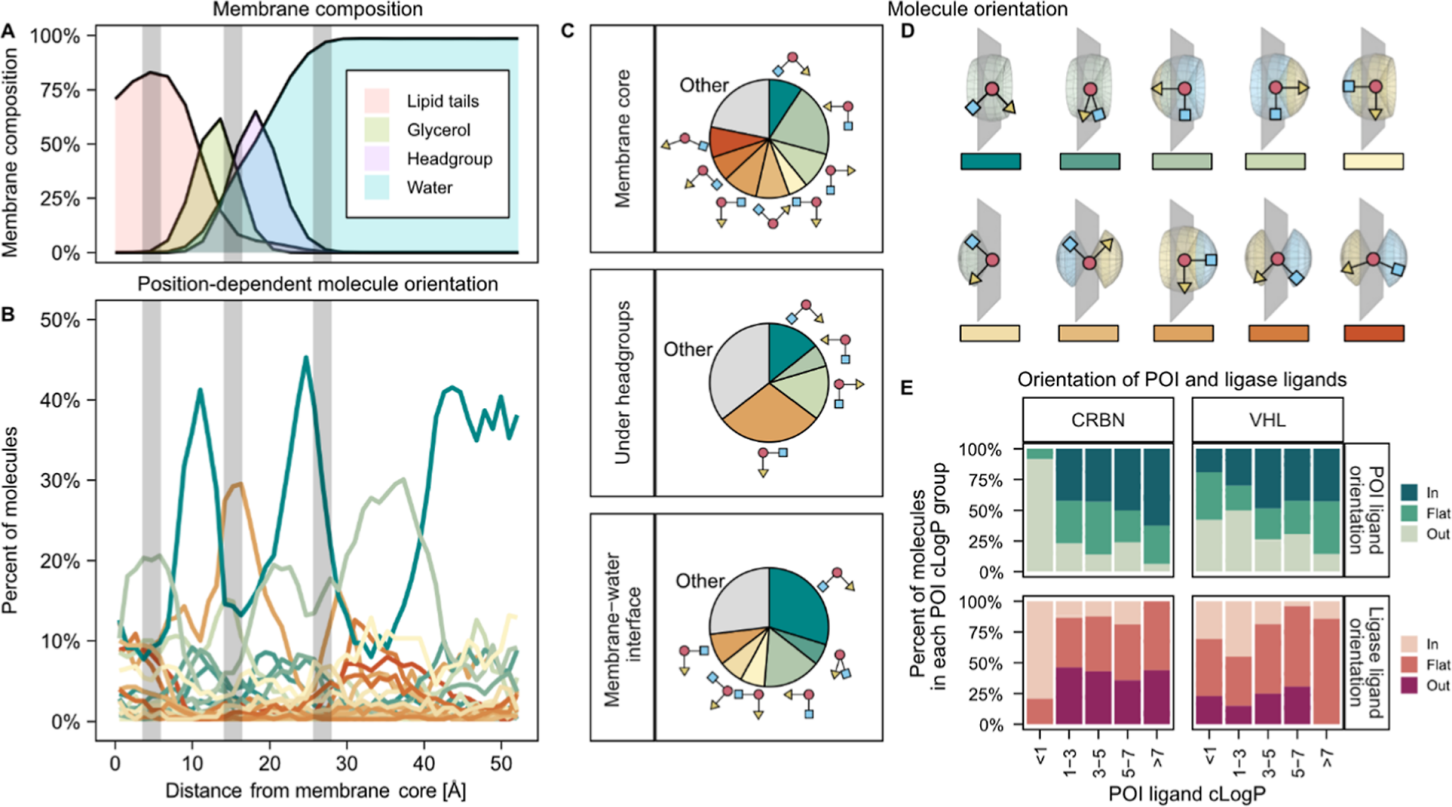
Orientations of representative PROTACs (*n* = 210 discrete molecules; 351 stereoisomers) in simulated phospholipid membranes. (A) Membrane composition. (B) Orientations of PROTACs at different depths of the membrane. Colors represent different shapes and orientations of PROTACs relative to the membrane, as indicated in D. (C) Molecular orientation at the membrane core, under the headgroups and at the membrane–water interface. (D) Schematic PROTAC orientations. Gray area indicates the membrane slice. (E) Orientation of POI and E3 ligands at the membrane core. Molecules were divided into CRBN- and VHL-based PROTACs and grouped based on POI ligand lipophilicity. The orientations of the POI and E3 ligand were assessed in each group separately.

At the water–membrane interface, the PROTACs tended to orient themselves flat against the membrane surface, while when positioned immediately beneath the headgroups, the E3 ligand more commonly pointed out toward the water, in particular for CRBN-directed PROTACs. Within the lipid tail region of the membrane, the dominating orientation was with the POI ligand pointing toward the core. This is reasonable, given that the POI ligand often is the most lipophilic part of the PROTAC and thus is expected to interact the most with the phospholipid tails.

Compounds with low penetration barriers at the membrane core (cluster A; Figure 4E) more often exhibited stretched conformations, with the POI ligand pointing toward the membrane center and the E3 ligand pointing toward the water, whereas in the cluster with higher membrane barriers (cluster C), the E3 ligand more commonly pointed inward to the membrane center (Figure S8). Interestingly, when the PROTAC centers-of-mass were positioned in bulk water at a distance of ∼35 Å from the membrane core, the dominating orientation was with the POI ligand toward the membrane, suggesting a preference for initiating interactions with the phospholipid headgroups through their POI ligand domain (Figure 5B).

Clear trends in molecule orientation were observed depending on POI ligand lipophilicity, with high-lipophilicity POI ligands more often pointing toward the membrane center and PROTACs with more hydrophilic POI ligands more often orienting their E3 ligase ligand toward the membrane center (Figure 5E). These effects were both more pronounced for CRBN-binding PROTACs than for VHL-binding ones, likely due to the larger difference between the E3 ligand and POI ligand lipophilicity for the former PROTAC class.

Assessing the exposed surface polarity for the conformational ensembles at different depths in the model lipid membrane revealed a more diverse picture. While the majority (54%) of the studied molecules predominantly sampled lower-polarity conformations in the membrane core than in water (with up to 102 Å^2^ reduction in exposed PSA; Table S3), a substantial proportion of the compounds showed the opposite behavior, with 11% of all compounds exposing 10 Å^2^ or more excess PSA in the hydrophobic membrane environment than in water. Notably, however, more than 90% of the molecules exposing more polar surface in the membrane core also had a correspondingly increased total exposed surface area. This indicates that such molecules adopt more stretched conformations in the membrane core, exposing more polarity, but at the same time also exposing more nonpolar atoms, compensating for the desolvation penalty.

### Membrane Interactions in Defined Structure Series

Our global analyses thus indicated that PROTAC–membrane interactions are influenced by both E3 ligand and linker properties, suggesting opportunities to optimize PROTACs for a given protein of interest by modifying these functional units. To confirm these findings and assess membrane affinity-driving features more systematically, we designed and synthesized a series of new PROTAC molecules, incorporating identical POI ligands but with variations in the E3 ligand moiety and linker length and chemistry (Figure 6A), and measured their affinities to the cell homogenate through equilibrium dialysis. The choice of linkers was partly based on emerging data of clinical PROTACs, which tend to have shorter, ring-containing linkers. Biological relevance was confirmed by E3 ligase recruitment in HEK293 cells (data not shown). This expanded set confirmed the initial trend, with observations of increasing membrane partitioning for PROTACs with a lower energy minimum at the water–membrane interface and greater molecular lipophilicity (Figure S9). Exchanging the E3 ligand resulted in a 3-fold higher bound-to-unbound ratio of the VHL-directed **11** than of the POI-matched (and similarly linked) CRBN-directed **9** and a corresponding deeper energy minimum for **11**. Thus, the substitution of the E3 ligand strongly affects membrane affinity, most likely due to the difference in lipophilicity that follows. We also confirmed our previous findings that linker length and chemistry affect membrane partitioning and depth of the energy minima, with PROTACs containing alkane linkers exhibiting higher binding and deeper energy minima than structurally matched compounds containing equally long PEG-based linkers (Figure 6G).

**Figure 6:**
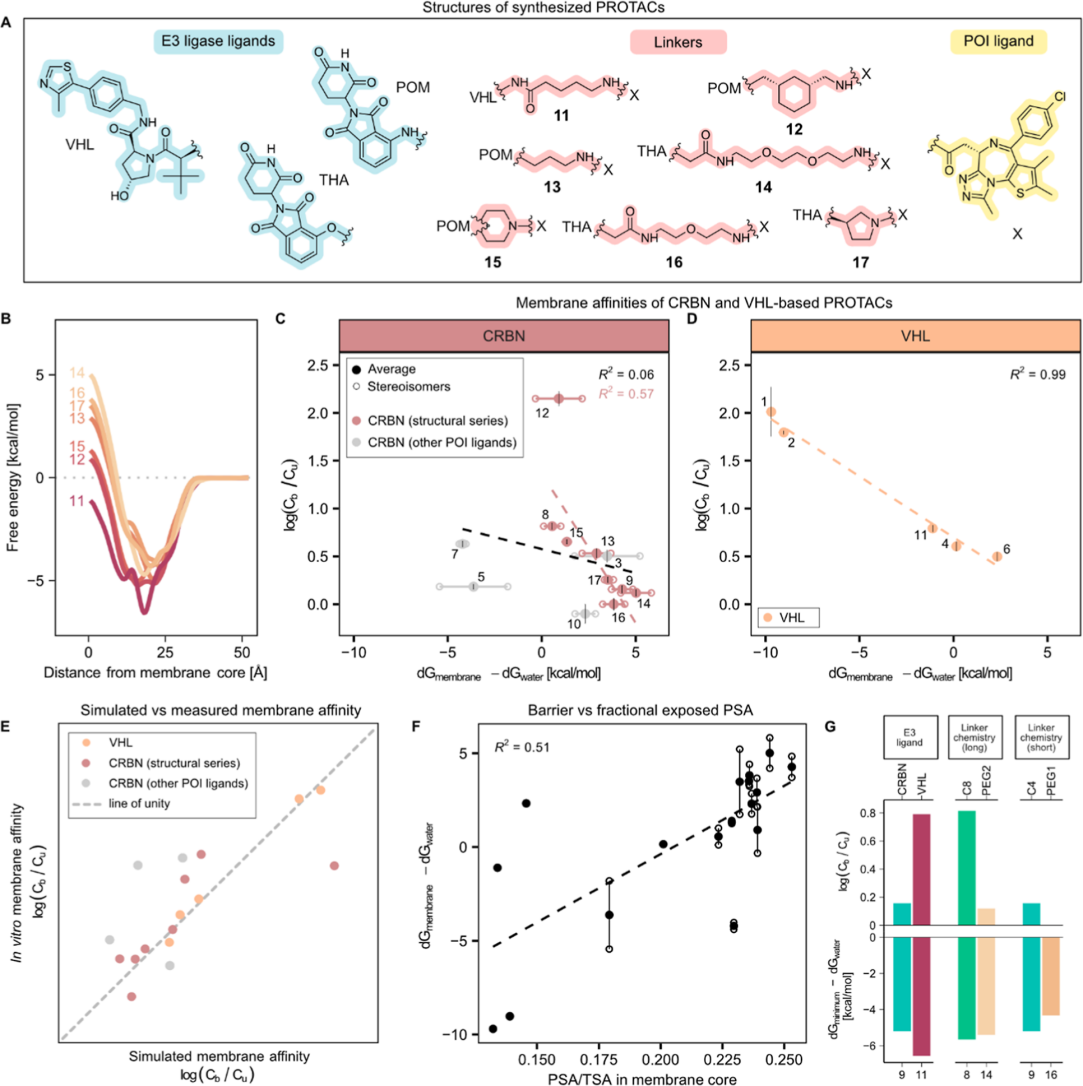
Cell membrane affinities of a new series of structurally related PROTACs. (A) Molecular structures of PROTACs **11**–**17**. (B) Average free energy profiles for compounds **11–17**. (C,D) Bound-to-unbound concentration ratios (mean ± SD, *n* = 3) correlate strongly to the water-to-membrane core energy barrier for VHL-binding PROTACs (panel D, orange full circles), while a more moderate correlation is seen for CRBN-binding PROTACs (panel C) that share the same E3 ligand and POI ligand. Full circles show the average of a 1:1 racemic mixture, and open circles show values for individual stereoisomers. PROTACs highlighted in red belong to a structural series with the identical E3 ligand and POI ligand, while gray circles show additional CRBN-binding PROTACs that are more structurally diverse. (E) Correspondence between simulated and in vitro measured membrane affinities. (F) Energy barrier water-to-membrane core correlates moderately to fraction solvent exposed polar surface area of total solvent exposed surface area. Full circles show the average of a 1:1 racemic mixture, and open circles show values for individual stereoisomers. (G) Comparison of energy barriers and experimental membrane binding. Top: Bound-to-unbound concentration ratios (mean). Bottom: Depth of energy minima. Left: The VHL-binding **11** has a lower energy minimum and higher cell homogenate binding than the CRBN-binding **9**. Middle: **8** with a long alkane linker has a lower energy minimum and higher experimental binding than **14** with an equally long PEG-linker. Right: **9**, with a shorter alkane linker, has a lower energy minimum and higher cell homogenate binding than **16** with a PEG-based linker.

Despite relatively small differences in the overall molecular structure, the new series of analogs varied widely in the height of the energy barrier at the membrane core—similar to observations for compounds **1**–**10** (Figures 2D and 6B). This energy barrier is associated with both membrane affinity and the rate of solute permeation (flip-flop) across the lipid bilayer.^57^ Notably, there was a clear correlation between the barrier height and in vitro membrane affinity for the VHL-directed PROTACs in our set. A similar trend was seen for the subset of CRBN-directed PROTACs that share the same POI ligand, whereas global trends in the full set of CRBN-directed PROTACs were obscured by the wide variation in the POI ligand properties (Figure 6C–E). Interestingly, throughout the VHL series, the E3 ligand remained identical, and all compounds but one (compound **11**) contained similar PEG-based linkers, whereas in the CRBN analogs, structural variations arise solely from the linker domain and the nitrogen versus oxygen connecting atom in pomalidomide/thalidomide-based E3 ligands. Thus, PROTAC–membrane affinities can be modulated by isolated variations in either the POI ligand or the linker regions, indicating that rational optimization of the linker region can be used to balance properties in the functionally active domains.

Analyzing the preferential orientation of the experimentally tested PROTACs, there was a clear tendency to orient the POI ligand toward the membrane center and the E3 ligand domain either toward the water phase or along the membrane surface. This is likely explained by the greater relative lipophilicity of the POI ligand. The trend is more pronounced in the CRBN-directed PROTACs, consistent with the greater difference in the lipophilicity between the E3 ligase and POI binding domains in these compounds.

Finally, compounds with proportionally greater exposed polar surface, in general, exhibited higher barriers at the membrane core (Figure 6F). The trend was more evident in CRBN-binding than in VHL-binding PROTACs, indicating that the ability to shield polarity is more important for the former and consistent with the less lipophilic nature of the CRBN ligands (Table 1). The E3 ligand of CRBN-binding PROTACs also more often pointed out toward the water, indicating a preference for interactions with the lipid headgroups. Flip-flop and permeation across the membrane can thus be assumed to be more difficult for this class of PROTACs, as a result of both an increased desolvation cost at the interface and a higher barrier across the membrane core.

## Conclusions

Through systematic experimental and computational analyses, we provide insights into how the E3 ligand, the linker chemistry, and general molecular characteristics affect the interactions of PROTACs with cell membranes—properties that ultimately determine their membrane permeability, pharmacologically active intracellular free exposure, and overall distribution to and binding and retention in peripheral tissues.

Previous analyses of conformational dynamics of linear and macrocyclic beyond-rule-of-5 drugs have indicated that polarity shielding is a major factor in determining cell permeability.^26,28–31^ We see similar global trends in PROTACs, with a lower exposed polar surface in nonpolar than in polar environments, based on conformations sampled using molecular mechanics. Further, using quantum-mechanics-based calculations to delineate PROTAC interactions with simulated lipid membranes, we see that a majority of all compounds analyzed sample conformations with lower exposed polarity in the membrane core than in the water phase. At the same time, many PROTACs, when located in the core of the lipid membrane, preferentially orient in extended conformations pointing along the membrane normal, which in several cases led to greater average exposed polarity in the membrane core than in water. That said, compounds with such behavior typically also exposed a higher total surface area, and thus, the energy expense of exposing polar atoms in a nonpolar environment is compensated by an increased surface hydrophobicity.

Conformation-dependent shielding of the polar surface area was linked more strongly to the overall folding of the molecule, rather than the folding of the linker region alone. Thus, conformational rearrangement within the POI ligand and/or E3 ligand domains contributes to the observed polarity shielding. We note that there are clear differences between different linker types in the propensity of the PROTACs to form folded structures, which suggests that the choice of the linker plays an important role in the overall molecule folding. Long PEG-based linkers, with or without ring systems in the linker, resulted in the most folded molecules and are thus assumed to reduce exposed polar surface area the most. The caveat to this assumption is the greater polarity of the PEG linkers themselves, which tend to remain solvent-exposed also in folded structures.

An increase in the conformation-independent property cLogP, as well as in experimentally measured cell membrane binding, was associated with deeper energy minima at the membrane–water interface, while lower solvent exposed polar surface area was linked to a decreased energy barrier in the transition from the energy minima to the membrane core. These results suggest that the interactions of PROTACs with the lipid membrane are driven largely by the overall molecular lipophilicity and are compensated by the ability to shield polar functionality. Such environment-dependent polarity shielding appears more pronounced for CRBN-binding than for VHL-binding PROTACs and may partly compensate for the more polar nature of the CRBN ligands.

PROTACs with PEG-based linkers exhibited higher energy barriers at the membrane core, both in the global analyses of chemically diverse PROTACs and in defined structural series, while alkane-based linkers instead reduced this barrier. Different membrane interactions were also observed for VHL- and CRBN-binding PROTACs, where the former, in general, had smaller energy barriers in the membrane core.

Our measurements of passive permeability across Caco-2 cell monolayers indicated that lower energy barriers at the membrane core were associated with more favorable permeability, but since the barrier reduction is linked to an increased molecular lipophilicity, aqueous solubility may be correspondingly reduced. Furthermore, the more lipophilic PROTACs tended to yield deeper energy minima at the membrane–water interface, which in our experiments also corresponded to reduced permeability because of the increased energy penalty for redistributing to water.

In summary, an intricate picture emerges, in which PROTAC membrane affinity and permeability are affected by overall molecular properties and also by specific features of the constituent parts and by the conformations sampled in different molecular environments. We find that conformational flexibility is beneficial for PROTAC–cell membrane interactions by enabling environment-dependent shielding of polarity but is contrasted with the preference for extended conformations within the membrane. It thus appears that shorter-range intramolecular interactions leading to selective polarity shielding within the POI ligand and/or E3 ligand domains may be preferred over more drastic molecular folding in order to design PROTACs with improved interactions with cell membranes. Furthermore, our results suggest that PROTAC cell permeability can be limited either by high barriers against traversing the membrane core or by deep energy minima at the membrane–water interface and that generic increases in compound lipophilicity therefore may not lead to the intended increases in permeability. However, our analyses of the individual contributions from E3 ligase ligands, linkers, and POI ligands to PROTAC membrane affinities and permeation barriers support that suboptimal properties in one component can be compensated by optimizing the other structure units.

## Experimental Section

### Calculation of 2D Descriptors

SMILES codes from the original data sources or manually sketched molecules were converted to the canonical format using OpenBabelGUI (v 3.1.1).^61^ E3 ligands were identified through a visual inspection of the molecules, as were the POI ligands. All molecules were then decomposed into their constituent E3 ligand, linker, and POI ligand domains using a custom RDKit (v 2021.09.2) script implemented in Python (v 3.9.7).^62,63^ 2D descriptors, including molecular weight (MW), calculated Log *P* (*c* Log *P*), hydrogen bond acceptor and donor counts (HBA/HBD), number of freely rotatable bonds (nRotB), and topological polar surface area (TPSA), were calculated on unprotonated molecules using the module Chem. Descriptors in RDKit for the intact molecule as well as for the separated structural domains. With the exception of *c* Log *P*, these descriptors are all fully additive; minute deviations from additivity in the atom-based *c* Log *P* model used were deemed acceptable for the purpose of analyzing lipophilicity contributions from the constituent parts of PROTACs. The most common linker patterns were identified and matched to all molecules in the database by using RDKit. The identified linkers were grouped based on whether or not they contained ring motifs and on the chemistry and length of linear linkers.

### Principal Component Analysis (PCA)

To ensure selection of a diverse and representative subset of molecules from the large database, a PCA of the calculated 2D descriptors (MW, *c* Log *P*, HBA, HBD, nRotB, and TPSA) was performed in R,^64^ using the function prcomp from the stats package.

### Conformational Sampling

3D starting geometries were prepared from SMILES strings in LigPrep (Schrödinger Release 2021-3: LigPrep, Schrödinger, LLC, New York, NY, 2021) using the OPLS4 force field.^65^ Molecules were desalted, tautomers were generated, and previously specified chiralities were retained. Tautomeric variants were extremely rare, and hence, no specific analyses were performed on these. Stereoisomery of nondefined stereocenters were varied for all molecules, and ionization states relevant at a pH of 7.0 + 0.2 were generated for 400 of the 911 molecules. An un-ionized state was always generated. A total of 2359 molecular variants were generated and subsequently analyzed.

Conformational sampling of the generated variants was performed in water and chloroform using MacroModel (Schrödinger Release 2021-3: MacroModel, Schrödinger, LLC, New York, NY, 2021) and the OPLS4 force field. Default settings were applied if not otherwise stated. Minimization was performed using the Polak–Ribier Conjugate Gradient (PRCG) method with a maximum of 5000 iterations per molecule. Minimizations were set to converge on gradients with a threshold of 0.05. The conformational sampling used the Mixed torsional/Low-mode sampling method with an automatic setup. An intermediate torsion sampling option was used. Mirror-image conformations were retained, and the maximum number of steps was set to 5000, with 100 steps per bond. The energy window for saving structures was set to 21.0 kJ/mol, and redundant conformers were eliminated using a maximum atom deviation of 0.5 Å. Molecule variants containing negatively charged sulfonamides were not possible to process in the chloroform environment and were thus excluded from further analysis.

### Calculation of 3D Descriptors

A custom PyMol (v 2.1) script^29^ was used to calculate van der Waals and solvent-accessible (rolling water probe) polar and total surface areas (PSA/TSA) of each of the conformations generated in the conformational sampling.

Conformational flexibility in the linker region and in the full molecule were calculated by identifying the centers of mass of the E3 ligand and POI ligand domains, as well as the anchor points for the linker, in both 2D and 3D structures. The 2D structures used were generated in RDKit from SMILES strings, and the 3D structures were the conformers generated in the conformational sampling.

The maximum distance between E3 ligand and POI ligand centers of mass (representing the distance in a fully stretched molecule) was calculated from the 2D structures by taking the sum of the distances between the E3 ligand center of mass and E3 ligand–linker anchor point (*d*_E3 ligand, 2D_), E3 ligand–linker anchor point and POI ligand linker anchor point (*d*_linker, 2D_), and POI ligand linker anchor point and POI ligand center of mass (*d*_POI ligand, 2D_) (eq 1).

1 $$d_{\max,2D}=d_{E3\;ligand,2D}+d_{linker,2D}+d_{POI\;ligand,2D}$$

The distance between the E3 ligand center of mass and POI ligand center of mass (*d*_E3 ligand to POI ligand, 3D_) was calculated for each 3D conformer and compared to the maximum distance, giving the measure “molecule contraction” (eq 2).

2 $$molecule\;contraction=\frac{d_{E3\;ligand\;to\;POI\;ligand,3D}}{d_{\max,2D}}\times 100$$

Analogously, the degree of linker stretch was calculated for each 3D conformer and compared to the maximum linker length (“linker contraction”) (eq 3).

3 $$linker\;contraction=\frac{d_{linker,3D}}{d_{linker,2D}}\times 100$$

### Molecular Dynamic Simulation of the DMPC Membrane Model

A membrane model was simulated consisting of DMPC lipids. The system was simulated at 303 K, using 70 water molecules per lipid. The atomistic molecular dynamics (MD) simulations were performed in the GROMACS software package (version 2021.3).^66–72^

A pre-equilibrated DMPC bilayer was obtained,^73,74^ containing 128 DMPC lipid molecules as well as water. The temperature-independent paramagnetism 3p water model was used according to literature recommendations.^75^ To ensure enough room for the PROTACs in the solvent phase, the box size was adjusted in the *z* dimension from 62 Å to 108 Å and additional solvent was added with a concentration of 0.15 M NaCl using GROMACS and a perl script (http://www.mdtutorials.com/gmx/membrane_protein/Files/water_deletor.pl).

Energy minimization of the lipid membrane was performed using a steepest descent algorithm with 50,000 steps. Long-range electrostatic interactions were calculated using the particle-mesh Ewald method^76^ with a cutoff of 1.4 nm. van der Waals interactions were treated with a plain cutoff at 1.4 nm.^77^ Long-range dispersion corrections for energy and pressure were added.^73^ Periodic boundary conditions were imposed in every dimension.

The simulation was run in semi-isotropic conditions where pressure in the xy plane was coupled separately from the *z* direction. Pressure was kept constant at 1 bar using the Parrinello–Rahman barostat,^78^ with a coupling constant of 10.0 ps and a compressibility of 4.5 × 10^–5^ bar^–1^. The temperature was kept constant at 303 K using the Nosé–Hoover thermostat,^79,80^ coupling the membrane and solvent separately, with a coupling constant of 0.5 ps. The P-LINCS algorithm was used to constrain all covalent bonds in the lipids,^81^ and for water, the SETTLE method was applied,^82,83^ allowing for a time step of 2 fs, enforced using the leapfrog integrator.^84^

The membrane was equilibrated in two steps, first with an *NVT* (number of particles, volume, and temperature) ensemble for 10 ns with a 2 fs time step, followed by an *NPT* (number of particles, pressure, and temperature) ensemble for 40 ns. Pressure, temperature, density, and box vectors were verified to have stabilized around desired values, with no trends. Production simulations were run for 100 ns with a time step of 2 fs. Coordinates were saved every 1 ps, and the neighbor list was updated every 10th step.

### COSMO Calculations

In COSMOtherm simulations, statistical thermodynamics were applied to surface polarization charge densities obtained from density functional theory (DFT) optimizations^51,52,55^ separately performed for each component of the simulation, i.e. water, the sodium cation, the chloride anion, a representative DMPC lipid, and a PROTAC molecule in its un-ionized state. A representative lipid conformation was selected in a multistep process. First, the lipid membrane MD frame with the area per lipid most closely representing the average was selected. All 128 lipids in this frame were subjected to DFT/COSMO calculations in the COSMOconf. From these, the RMSD compared to the conformers observed in the MD simulation was calculated using GROMACs tool gmx rms, using the trajectory from the MD simulations as the reference. The most representative (lowest RMSD) low-energy conformation was selected for further calculations in COSMOtherm. Notably, COSMOtherm is robust to the choice of the lipid conformer, as long as it is fairly representative.^52^ The BP-TZVP basis set was used, and DFT optimizations were performed in COSMOconf (Biovia COSMOlogic v 21.0) to obtain sigma profiles.

Six frames from the equilibrated DMPC membrane were separately imported into COSMOtherm (Biovia COSMOlogic v 21.0) as a planar membrane and divided into 50 slices of approximately 0.1 nm thickness, as recommended in the literature.^52^ Each slice was treated as a homogeneous phase with its own sigma profile. Input files for water, the Na cation, and the Cl anion were used as supplied with the COSMO software suite and were added to the membrane file together with the selected DMPC lipid conformer. Un-ionized and ionized PROTAC molecules were then added to the system and rotated in 162 different orientations in each layer using the COSMOtherm plugin COSMOmic to generate layer-dependent solvation energies with BP_TZVP_24 or BP_TZVP_ELYTE parametrization, respectively. Local sigma profiles were calculated for each layer, taking into account the fact that PROTACs due to their size may extend across multiple layers, and an integral sigma profile was generated. From this, the solvation energy for each molecule in each layer was calculated, and a free energy profile was generated.

### Hierarchical Clustering

To identify molecules with similar membrane interaction behavior, free energy profiles generated from the combined conformer sets were hierarchically clustered using the hclust function with the complete linkage method in the R Stats Package in R (v.4.2.2).^64^

### Calculation of the Conformational Ensemble

To elucidate the conformation-dependent free energy of solvation of the studied PROTACs at different positions in a phospholipid membrane, free energy profiles from combined conformer sets for each molecule, as well as for their individual conformers, were calculated at 310 K in DMPC membranes using COSMOtherm.^51^ Free energy calculations were repeated using each of the six snapshots captured over the MD simulation time frame. To allow averaging of the free energy profiles at identical positions along the membrane normal, the profiles were linearly interpolated using R, and then, average free energy profiles were calculated for each conformer set or conformer. The profiles for the separated conformers were Boltzmann weighted using a custom *R* script to derive the highest-probability conformational ensemble in each section of the membrane.

### Calculation of Molecule Orientation

To assess the preferential orientation and bending of PROTAC molecules at various positions in the DMPC/water system, the molecules were separated in their E3 ligand, linker, and POI ligand domains, and the relative positions of the respective weighted centers of mass (hydrogens excluded) were determined. The molecule was kept at a fixed orientation in COSMOtherm, and the membrane was rotated to find the lowest energy orientation. To allow comparison among conformations, the coordinate systems were first realigned. The most probable orientation of each molecule was then identified at different distances from the membrane core. First, the linker center of mass was aligned to the origin. Then, three separate angles were calculated: (1) between the membrane normal and linker to the E3 ligand vector, (2) between the membrane normal and the linker to the POI ligand vector, and (3) between the linker to the E3 ligand vector and linker to the POI ligand vector. Each type of angle could take a value of 0° to 180° and was split in three 60° sections. This generated 27 different groups, to which each molecule was assigned at every section of the membrane.

### Determination of Fraction Unbound Drug in the Cell Homogenate

Reagents and cell culture media were obtained from Sigma-Aldrich (St. Louis, MO) or Invitrogen (Carlsbad, CA). Commercially available PROTACs were obtained from Tocris (Bristol, UK), new PROTACs were synthesized by SciLifeLab (Solna, Sweden), and small molecule drugs were obtained from Sigma-Aldrich (St Louis, MO) and were of ≥95% purity. Stock solutions of compounds (10 mM) were prepared in dimethyl sulfoxide (DMSO) and stored aliquoted at −80 °C. Reagent tubes and plates were made with Eppendorf Protein LoBind (Hamburg, Germany).

The unbound fraction of a selected series of PROTACs was determined through equilibrium dialysis against the cell homogenate. HEK293 cells (ATCC, Manassas, VA) were grown in Dulbecco’s modified Eagle’s medium (DMEM) supplemented with 10% fetal bovine serum (FBS) at 37 °C, 95% relative humidity, and 5% CO_2_. Cell pellets were harvested using 0.05% trypsin and centrifuged at 260*g* for 5 min. The cells were resuspended in Dulbecco’s phosphate buffer (DPBS), counted using a Luna II cell counter (Logos Biosystems, Anyang, South Korea) and centrifuged at 260*g*. The supernatant was discarded, and cell pellets were stored at −80 °C prior to cell homogenate preparation. On the day of the experiment, cell pellets were thawed and resuspended in Hank’s balanced salt solution (HBSS) buffered with 20 mM HEPES (pH 7.4) to a concentration of 10 × 10^6^ cells/mL. The cell suspension was homogenized using ultrasonication for 10 s at 20% intensity while kept on ice.

Binding to the cell homogenate was determined at 0.1 μM for all compounds. A Rapid Equilibrium Dialysis device (Thermo Fisher, Waltham, Massachusetts) was used to dialyze the samples by adding 200 μL of the compound-spiked cell homogenate to one chamber and 350 μL of HBSS/HEPES to the other chamber. The cassette was incubated on an Eppendorf ThermoMixer C (Hamburg, Germany) at 900 rpm for 4 h at 37 °C.

Compound stability was assessed by adding the compound-spiked cell homogenate to an empty well in the dialysis cassette. At the end of the experiment, these concentrations were compared to identical control samples stored at 4 °C. Mass balance was calculated at the end of each experiment. Further, in all wells, two control compounds (atorvastatin and lopinavir) were included to ensure that the assay plate was functional. All compounds were tested in triplicate in one experiment. Compound **2** was only detected on the cell homogenate side, and thus, the concentration on the buffer side was set to half of the lower limit of quantification to allow calculation of an approximate cell homogenate binding.

Final samples were matrix matched by taking 1 part sample and 1 part blank cell homogenate or buffer. Proteins were precipitated using 1 part matrix matched sample and 3 parts acetonitrile supplemented with 0.3% formic acid (FA) and 5 nM verapamil (internal standard), and this was diluted 1:1 with Milli-Q prior to centrifugation at 2500*g* for 20 min. Compound concentrations in the supernatant were quantified using a reversed phase system with a Waters Acquity UPLC HSS T3 column (50 × 2.1 mm, 1.8 μm) in an Aquity UPLC connected to a Xevo TQ-XS mass spectrometer (Waters Corp., Milford, MA). The chromatographic run was done using solvent A (0.1% FA in Milli-Q) and solvent B (0.1% FA in acetonitrile Optima LC/MS grade (Fisher Scientific)) with isocratic conditions between 0 and 0.3 min (99.8% solvent A and 0.2% solvent B), a linear gradient between 0.3 and 1.3 min (from 0.2% to 95% solvent B), isocratic conditions between 1.3 and 1.8 min (95% solvent B), and equilibration back to start conditions between 1.8 and 2.3 min. Mass transitions, cone voltages, and collision energies can be found in Table S4.

### Caco-2 A-B Permeability with Preincubation

Transepithelial transport of the compounds through a Caco-2 monolayer (ATCC, HTB-37) was determined in 14–18 days old monolayers in Costar 24-well cell culture cluster plates (polycarbonate membrane, 0.4 μm pore size). Chamber volumes were 200 and 800 μL on the apical and basolateral sides of the cell monolayers, respectively, and all incubations were performed with prewarmed buffers in an orbital shaking incubator at 480 rpm and 37 °C. Prior to the assay, cells were washed with HBSS supplemented with 25 mM HEPES (HBSS-HEPES), pH 7.4, to remove the culture medium. After 15 min of equilibration, the transepithelial electrical resistance (TEER) was determined to assess acceptance of the cell plates into the assay. A second measurement and a lucifer yellow leakage determination was carried out after performing all the transport experiments to monitor integrity of the cell monolayers throughout the study.

Before the permeability study, to minimize nonspecific binding of PROTACs to the cells and plates, the cells were preincubated with 10 μM compound solution on the apical (donor) side for 1 h, analogous to Muschong et al.^85^ Then, cells were washed twice with HBSS-MES pH 6.5 containing 1% BSA. Then, permeability in the absorptive direction (A–B, apical-to-basolateral) was studied over 120 min at a 10 μM test compound concentration. The compound solutions were freshly prepared from DMSO stock solutions diluted into HBSS supplemented with 25 mM MES, pH 6.5, containing efflux inhibitors (50 μM quinidine, 30 μM benzbromarone, and 20 μM sulfasalazine); the final solvent concentration was always 0.5%.

Samples from the donor side (10 μL) were drawn immediately after the addition of the test compound and after 45 and 120 min. The donor samples were diluted 1:10 in HBSS. From the receiver compartment, an amount of 100 μL was withdrawn after 45 and 120 min and replaced with fresh HBSS-HEPES, pH 7.4.

Upon completion of the study, all samples were quenched with 300 μL of acetonitrile and analyzed subsequently using UPLC/MS/MS. The permeability was determined as the appearance rate of the compound on the receiver side, in relation to donor concentration, according to eq 4

4 $$P_{app}=\frac{dQ}{\left[\left(dt\right)\left(T\right)\right]}\times \frac{A}{D}$$

where d*Q*/[(d*t*)­(*T*)] is the slope of the permeation profile across the Caco-2 cell monolayers, *A* is the surface area of the Transwell insert (0.33 cm^2^), and *D* is the concentration on the donor side. Following the permeability experiments, recovery from the donor and receiver compartments was calculated using eq 5

5 $$recovery\;\left(\%\right)\;\left(0-120\;min\right)=\frac{C_{R}^{120}\times V_{R}+C_{R}^{45}\times 0.1+C_{D}^{45}\times 0.01+C_{D}^{120}\times V_{D}}{D_{0}\times V_{D}}\times 100$$

### General Chemistry Methods

All solvents and reagents were used as received from commercial suppliers. Column chromatography was employed on normal-phase silica gel (40–60 μm and 60 Å). ^1^H nuclear magnetic resonance (NMR) and ^13^C NMR spectra were recorded on a 400 MHz spectrometer at 298 K and calibrated using the residual peak of the solvent as an internal standard: CDCl_3_ (δ_H_ 7.26 ppm, δ_C_ 77.16 ppm), CD_3_OD-*d*4 (δ_H_ 3.31 ppm, δ_C_ 49.00 ppm), and DMSO-*d*_6_ (δ_H_ 2.50 ppm, δ_C_ 39.52 ppm). NMR spectra are reported as follows: chemical shift, multiplicity (s = singlet, d = doublet, t = triplet, q = quartet, m = multiplet, dd = doublet of doublets), coupling constant (*J*) in Hertz (Hz) (if applicable), and integration (proton spectra only). NMR spectra are listed in the Supporting Information. Analytical liquid chromatography coupled with mass spectrometry (LCMS) was performed using the column ACE 3 C8 (50 × 3.0 mm); water (0.1% TFA) and acetonitrile were used as mobile phases at a flow rate of 1 mL/min, with a gradient time of 3.0 min. High-resolution mass spectra were recorded on a QTOF mass spectrometer with electrospray ionization (VIP-HESI). Ions were detected in the positive mode. ESI-L Low Concentration Tuning Mix was used for instrument calibration prior to measurements. Samples were prepared as 1 μM solutions in ACN/water 1:1 v/v and directly infused into the mass spectrometer at a constant rate of 10 μL/min. All final compounds were assessed to be >95% pure by LCMS analysis (UV detection was made at 254 and 305 nm). HPLC traces can be found in the Supporting Information.

#### Synthesis of (2*S*,4*R*)-1-((2*S*)-2-(5-(2-((6*S*)-4-(4-Chlorophenyl)-2,3,9-trimethyl-6*H*-thieno­[3,2-*f*]­[1,2,4]­triazolo­[4,3-*a*]­[1,4]­diazepin-6-yl)­acetamido)­pentanamido)-3,3-dimethylbutanoyl)-4-hydroxy-*N*-(4-(4-methylthiazol-5-yl)­benzyl)­pyrrolidine-2-carboxamide (**11**)

DIPEA (17 μL, 0.1 mmol) was added to a stirred solution of JQ-1 carboxylic acid (10 mg, 0.025 mmol) and TBTU (8.4 mg, 0.026 mmol) in DMF (0.5 mL). After 30 min, **S-1** (13 mg, 0.025 mmol) was added, and the reaction was allowed to perform for 2 h. The mixture was diluted with EtOAc, washed with NaHCO_3_ sat. aq., dried over sodium sulfate, filtered, and concentrated to dryness. The resulting material was purified using automated flash chromatography (12 g silica cartridge, DCM/MeOH: 0–10% over 7 column volumes) to provide compound **11** (16 mg, 70% yield). ^1^H NMR (400 MHz, DMSO-*d*_6_): δ 8.98 (s, 1H), 8.56 (t, *J* = 6.1 Hz, 1H), 8.18 (t, *J* = 5.5 Hz, 1H), 7.88 (d, *J* = 9.4 Hz, 1H), 7.57–7.34 (m, 8H), 5.13 (d, *J* = 3.6 Hz, 1H), 4.55 (d, *J* = 9.5 Hz, 1H), 4.50 (dd, *J* = 8.4, 5.9 Hz, 1H), 4.43 (ddd, *J* = 10.1, 6.7, 3.1 Hz, 2H), 4.38–4.32 (m, 1H), 4.21 (dd, *J* = 15.9, 5.6 Hz, 1H), 3.71–3.61 (m, 2H), 3.25 (dd, *J* = 15.1, 8.3 Hz, 1H), 3.21–3.00 (m, 3H), 2.59 (s, 3H), 2.44 (s, 3H), 2.41 (s, 3H), 2.32–2.24 (m, 1H), 2.21–2.09 (m, 1H), 2.08–1.98 (m, 1H), 1.90 (ddd, *J* = 12.9, 8.6, 4.6 Hz, 1H), 1.62 (s, 3H), 1.65–1.37 (m, 4H), 0.93 (s, 9H). ^13^C NMR (101 MHz, DMSO-*d*_6_): δ 171.9 (2C), 169.7, 169.3, 163.0, 155.1, 151.3 (visible via HSQC coupling), 149.8, 147.7, 139.5, 136.8, 135.2, 132.3, 131.1, 130.7, 130.1 (2C), 129.8, 129.6, 129.6, 128.6 (2C), 128.5 (2C), 127.4 (2C), 68.9, 58.7, 56.4, 56.3, 53.9, 41.6, 38.4, 37.9, 37.6, 35.2, 34.6, 28.9, 26.4 (3C), 23.0, 15.9, 14.1, 12.7, 11.3. HRMS, ESI^+^ *m*/*z*: calcd for C_46_H_54_ClN_9_O_5_S_2_ [M + H]^+^, 912.3451; found, 912.3447.

#### 2-((6*S*)-4-(4-Chlorophenyl)-2,3,9-trimethyl-6*H*-thieno­[3,2-*f*]­[1,2,4]­triazolo­[4,3-*a*]­[1,4]­diazepin-6-yl)-*N*-(((1*RS*,3*SR*)-3-(((2-(2,6-dioxopiperidin-3-yl)-1,3-dioxoisoindolin-4-yl)­amino)­methyl)­cyclohexyl)­methyl)­acetamide (**12**)

DIPEA (17 μL, 0.1 mmol) was added to a stirred solution of JQ-1 carboxylic acid (10 mg, 0.025 mmol), **S-2** (9.9 mg, 0.025 mmol), and TBTU (8.4 mg, 0.026 mmol) in DMF (0.5 mL). The reaction was allowed to perform at room temperature overnight. The mixture was diluted with EtOAc, washed with NaHCO_3_ sat. aq., dried over sodium sulfate, filtered, and concentrated to dryness. The resulting material was purified using automated flash chromatography (12 g silica cartridge, DCM/MeOH: 0–10% over 7 column volumes) to provide compound **12** (12.5 mg, 65% yield). ^1^H NMR (400 MHz, DMSO-*d*_6_): δ 11.09 (s, 1H), 8.19 (q, *J* = 6.4 Hz, 1H), 7.56 (ddd, *J* = 9.4, 7.0, 2.5 Hz, 1H), 7.51–7.39 (m, 4H), 7.12–7.04 (m, 1H), 7.01 (d, *J* = 7.0 Hz, 1H), 6.63–6.52 (m, 1H), 5.05 (ddd, *J* = 12.9, 5.4, 1.8 Hz, 1H), 4.50 (dd, *J* = 8.4, 5.8 Hz, 1H), 3.35–3.10 (m, 4H), 3.08–2.80 (m, 3H), 2.64–2.50 (m, 5H), 2.41 (s, 3H), 2.08–1.97 (m, 1H), 1.85–1.69 (m, 4H), 1.66–1.55 (m, 4H), 1.55–1.40 (m, 1H), 1.28–1.14 (m, 1H), 0.97–0.78 (m, 2H), 0.66 (p, *J* = 12.7, 12.3 Hz, 1H). ^13^C NMR (101 MHz, DMSO-*d*_6_): δ 172.8, 170.1, 169.4, 169.0, 167.3, 163.0 (d), 155.1, 149.8, 146.6, 136.7, 136.2, 135.2, 132.3, 132.1, 130.7, 130.1 (d, 2C), 129.8, 129.6, 128.4 (2C), 117.3 (d), 110.4, 109.0, 54.0, 48.5, 48.2 (d), 44.9 (d), 37.7 (d), 37.6 (d), 37.4 (d), 37.0 (d), 34.6, 31.0, 30.4, 30.2 (d), 25.0, 22.2, 14.1 (d), 12.7, 11.3. HRMS, ESI+ *m*/*z*: calcd for C_40_H_41_ClN_8_O_5_S [M + H]^+^, 781.2682; found, 781.2677.

#### 2-((6*S*)-4-(4-Chlorophenyl)-2,3,9-trimethyl-6*H*-thieno­[3,2-*f*]­[1,2,4]­triazolo­[4,3-*a*]­[1,4]­diazepin-6-yl)-*N*-(3-((2-(2,6-dioxopiperidin-3-yl)-1,3-dioxoisoindolin-4-yl)­amino)­propyl)­acetamide (**13**)

DIPEA (17 μL, 0.1 mmol) was added to a stirred solution of JQ-1 carboxylic acid (10 mg, 0.025 mmol), **S-3** (8.2 mg, 0.025 mmol), and TBTU (8.4 mg, 0.026 mmol) in DMF (0.5 mL). The reaction was allowed to perform at room temperature overnight. The mixture was diluted with EtOAc, washed with NaHCO_3_ sat. aq., dried over sodium sulfate, filtered, and concentrated to dryness. The resulting material was purified using automated flash chromatography (12 g silica cartridge, DCM/MeOH: 0–10% over 7 column volumes) to provide compound **13** (10.7 mg, 60% yield). ^1^H NMR (400 MHz, DMSO-*d*_6_): δ 11.09 (s, 1H), 8.32 (t, *J* = 5.9 Hz, 1H), 7.56 (dd, *J* = 8.6, 7.0 Hz, 1H), 7.52–7.37 (m, 4H), 7.10 (d, *J* = 8.6 Hz, 1H), 7.02 (d, *J* = 7.0 Hz, 1H), 6.67 (td, *J* = 6.3, 2.1 Hz, 1H), 5.05 (dd, *J* = 12.8, 5.4 Hz, 1H), 4.52 (t, *J* = 7.2 Hz, 1H), 3.36 (q, *J* = 6.8 Hz, 2H), 3.29–3.16 (m, 4H), 2.88 (ddd, *J* = 17.8, 14.2, 5.7 Hz, 1H), 2.65–2.49 (m, 5H), 2.40 (s, 3H), 2.07–1.96 (m, 1H), 1.79–1.67 (m, 2H), 1.61 (s, 3H). ^13^C NMR (101 MHz, DMSO-*d*_6_): δ 172.8, 170.1, 169.7, 168.8, 167.3, 163.1, 155.1, 149.8, 146.3, 136.7, 136.2, 135.2, 132.3, 130.7, 130.1 (2C), 129.8, 129.6, 128.4 (2C), 117.2, 110.4, 109.2, 53.9, 48.5, 39.3, 37.8, 35.8, 35.8, 31.0, 28.9, 22.2, 14.0, 12.7, 11.3. HRMS, ESI+ *m*/*z*: calcd for C_35_H_33_ClN_8_O_5_S [M + H]^+^, 713.2056; found, 713.2052.

#### 2-((6*S*)-4-(4-Chlorophenyl)-2,3,9-trimethyl-6*H*-thieno­[3,2-*f*]­[1,2,4]­triazolo­[4,3-*a*]­[1,4]­diazepin-6-yl)-*N*-(2-(2-(2-(2-((2-(2,6-dioxopiperidin-3-yl)-1,3-dioxoisoindolin-4-yl)­oxy)­acetamido)­ethoxy)­ethoxy)­ethyl)­acetamide (**14**)

DIPEA (17 μL, 0.1 mmol) was added to a stirred solution of JQ-1 carboxylic acid (10 mg, 0.025 mmol), **S-4** (12 mg, 0.025 mmol), and TBTU (8.4 mg, 0.026 mmol) in DMF (0.5 mL). The reaction was allowed to perform at room temperature overnight. The mixture was diluted with EtOAc, washed with NaHCO_3_ sat. aq., dried over sodium sulfate, filtered, and concentrated to dryness. The resulting material was purified using automated flash chromatography (12 g silica cartridge, DCM/MeOH: 0–10% over 7 column volumes) to provide compound **14** (16 mg, 76% yield). ^1^H NMR (400 MHz, DMSO-*d*_6_): δ 11.11 (s, 1H), 8.26 (t, *J* = 5.7 Hz, 1H), 8.02 (t, *J* = 5.7 Hz, 1H), 7.80 (dd, *J* = 8.5, 7.3 Hz, 1H), 7.51–7.36 (m, 6H), 5.11 (dd, *J* = 12.9, 5.5 Hz, 1H), 4.79 (s, 2H), 4.50 (dd, *J* = 8.0, 6.3 Hz, 1H), 3.58–3.52 (m, 4H), 3.46 (dt, *J* = 10.5, 5.9 Hz, 4H), 3.36–3.16 (m, 6H), 2.89 (ddd, *J* = 17.8, 14.3, 5.8 Hz, 1H), 2.64–2.53 (m, 5H), 2.40 (s, 3H), 2.09–1.98 (m, 1H), 1.61 (s, 3H). ^13^C NMR (101 MHz, DMSO-*d*_6_): δ: 172.8, 169.9, 169.7, 166.9, 166.7, 165.4, 163.0, 155.1, 155.0, 149.8, 136.9, 136.8, 135.2, 133.0, 132.3, 130.7, 130.1 (2C), 129.8, 129.5, 128.4 (2C), 120.3, 116.8, 116.0, 69.6 (2C), 69.2, 68.8, 67.5, 53.8, 48.8, 38.6, 38.4, 37.5, 30.9, 22.0, 14.0, 12.7, 11.3. HRMS, ESI^+^ *m*/*z*: calcd for C_40_H_41_ClN_8_O_9_S [M + H]^+^, 845.2479; found, 845.2475.

#### 4-(4-(2-((6*S*)-4-(4-Chlorophenyl)-2,3,9-trimethyl-6*H*-thieno­[3,2-*f*]­[1,2,4]­triazolo­[4,3-*a*]­[1,4]­diazepin-6-yl)­acetyl)­piperazin-1-yl)-2-(2,6-dioxopiperidin-3-yl)­isoindoline-1,3-dione (**15**)

DIPEA (17 μL, 0.1 mmol) was added to a stirred solution of JQ-1 carboxylic acid (10 mg, 0.025 mmol), **S-5** (8.5 mg, 0.025 mmol), and TBTU (8.4 mg, 0.026 mmol) in DMF (0.5 mL). The reaction was allowed to perform at room temperature overnight. The mixture was diluted with EtOAc, washed with NaHCO_3_ sat. aq., dried over sodium sulfate, filtered, and concentrated to dryness. The resulting material was purified using automated flash chromatography (12 g silica cartridge, DCM/MeOH: 0–10% over 7 column volumes) to provide compound **15** (9.9 mg, 55% yield). ^1^H NMR (400 MHz, DMSO-*d*_6_): δ 11.10 (s, 1H), 7.75 (dd, *J* = 8.4, 7.1 Hz, 1H), 7.55–7.32 (m, 6H), 5.13 (dd, *J* = 12.8, 5.4 Hz, 1H), 4.61 (t, *J* = 6.8 Hz, 1H), 3.96–3.81 (m, 2H), 3.77–3.61 (m, 3H), 3.52–3.37 (m, 3H), 3.32–3.23 (m, 2H), 2.89 (ddd, *J* = 17.5, 14.3, 5.6 Hz, 1H), 2.65–2.52 (m, 5H), 2.42 (s, 3H), 2.10–2.00 (m, 1H), 1.64 (s, 3H). ^13^C NMR (101 MHz, DMSO-*d*_6_) δ: 172.8, 170.0, 168.5, 167.0, 166.4, 162.9, 155.2, 149.8, 149.4, 136.8, 136.0, 135.2, 133.6, 132.2, 130.7, 130.2 (2C), 129.9, 129.6, 128.5 (2C), 123.9, 117.0, 115.3, 54.2, 50.8, 50.4, 48.8, 45.0, 41.2, 34.8, 31.0, 22.0, 14.0, 12.7, 11.3. HRMS, ESI+ *m*/*z*: calcd for C_36_H_33_ClN_8_O_5_S [M + H]+, 725.2056; found, 725.2054.

#### 2-((6*S*)-4-(4-Chlorophenyl)-2,3,9-trimethyl-6*H*-thieno­[3,2-*f*]­[1,2,4]­triazolo­[4,3-*a*]­[1,4]­diazepin-6-yl)-*N*-(2-(2-(2-((2-(2,6-dioxopiperidin-3-yl)-1,3-dioxoisoindolin-4-yl)­oxy)­acetamido)­ethoxy)­ethyl)­acetamide (**16**)

DIPEA (17 μL, 0.1 mmol) was added to a stirred solution of JQ-1 carboxylic acid (10 mg, 0.025 mmol), **S-6** (10 mg, 0.025 mmol), and TBTU (8.4 mg, 0.026 mmol) in DMF (0.5 mL). The reaction was allowed to perform at room temperature overnight. The mixture was diluted with EtOAc, washed with NaHCO_3_ sat. aq., dried over sodium sulfate, filtered, and concentrated to dryness. The resulting material was purified using automated flash chromatography (12 g silica cartridge, DCM/MeOH: 0–10% over 7 column volumes) to provide compound **16** (13 mg, 65% yield). ^1^H NMR (400 MHz, DMSO-*d*_6_): δ 11.10 (s, 1H), 8.25 (t, *J* = 5.6 Hz, 1H), 8.04 (t, *J* = 5.6 Hz, 1H), 7.79 (dd, *J* = 8.6, 7.3 Hz, 1H), 7.52–7.33 (m, 6H), 5.11 (dd, *J* = 12.8, 5.3 Hz, 1H), 4.79 (s, 2H), 4.54–4.46 (m, 1H), 3.48 (dt, *J* = 11.3, 6.1 Hz, 4H), 3.38–3.17 (m, 6H), 2.88 (ddd, *J* = 17.6, 14.1, 5.6 Hz, 1H), 2.63–2.50 (m, 5H), 2.40 (s, 3H), 2.09–1.97 (m, 1H), 1.61 (s, 3H). ^13^C NMR (101 MHz, DMSO-*d*_6_): δ: 172.8, 169.9, 169.7, 166.9, 166.7, 165.4, 163.0, 155.1, 155.0, 149.8, 136.9, 136.7, 135.2, 133.0, 132.2, 130.7, 130.1 (2C), 129.8, 129.5, 128.5 (2C), 120.3, 116.7, 116.0, 69.0, 68.6, 67.5, 53.8, 48.8, 38.5, 38.4, 37.5, 30.9, 22.0, 14.0, 12.7, 11.3. HRMS, ESI^+^ *m*/*z*: calcd for C_38_H_37_ClN_8_O_8_S [M + H]^+^, 801.2217; found, 801.2215.

#### 4-((1-(2-((6*S*)-4-(4-Chlorophenyl)-2,3,9-trimethyl-6*H*-thieno­[3,2-*f*]­[1,2,4]­triazolo­[4,3-*a*]­[1,4]­diazepin-6-yl)­acetyl)­pyrrolidin-3-yl)­oxy)-2-(2,6-dioxopiperidin-3-yl)­isoindoline-1,3-dione (**17**)

##### *tert*-Butyl 3-((2-(2,6-Dioxopiperidin-3-yl)-1,3-dioxoisoindolin-4-yl)­oxy)­pyrrolidine-1-carboxylate (**S-9**)

*tert*-Butyl 3-hydroxypyrrolidine-1-carboxylate (**S-8**) (162 mg, 0.87 mmol) and triphenylphosphine (526 mg, 2 mmol) were added to a stirred solution of 2-(2,6-dioxopiperidin-3-yl)-4-hydroxyisoindoline-1,3-dione (**S-7**) (250 mg, 0.91 mmol) in THF (10 mL). DIAD (358 μL) diluted in THF (6 mL) was then added dropwise to the mixture, and the reaction was allowed to perform overnight at room temperature. The mixture was diluted with EtOAc, washed with water, dried over sodium sulfate, filtered, and concentrated to dryness. The resulting material was purified using automated flash chromatography (40 g silica cartridge, iso-Hexane/EtOAc, 1:3) to provide compound **S-9** (200 mg, 50% yield). ^1^H NMR (400 MHz, MeOD-*d4*): δ 7.80–7.71 (m, 1H), 7.49–7.38 (m, 2H), 5.26 (dd, *J* = 3.9, 2.0 Hz, 1H), 5.09 (dd, *J* = 12.4, 5.6 Hz, 1H), 4.75–4.45 (m, 1H), 3.71–3.49 (m, 4H), 2.94–2.80 (m, 1H), 2.80–2.63 (m, 2H), 2.28–2.15 (m, 2H), 2.13 (ddd, *J* = 10.3, 5.4, 3.1 Hz, 1H), 1.49–1.39 (m, 9H). LC–MS, ESI^+^ *m*/*z*: 344.1 [M + H-Boc]^+^.

#### 4-((1-(2-((6*S*)-4-(4-Chlorophenyl)-2,3,9-trimethyl-6*H*-thieno­[3,2-*f*]­[1,2,4]­triazolo­[4,3-*a*]­[1,4]­diazepin-6-yl)­acetyl)­pyrrolidin-3-yl)­oxy)-2-(2,6-dioxopiperidin-3-yl)­isoindoline-1,3-dione (**17**)

Step 1: HCl (4N, in 1,4-dioxane) (6 mL, 24 mmol) was added to a stirred solution of **S-9** (200 mg, 0.45 mmol) in 1,4-dioxane (4 mL). After 3 h, the heterogeneous solution was diluted with iso-hexane and filtered through a P3 glass-silica pad. The white solid was dried under vacuum to provide 2-(2,6-dioxopiperidin-3-yl)-4-(pyrrolidin-3-yloxy)­isoindoline-1,3-dione HCl salt (140 mg, 94% yield).
Step 2: DIPEA (17 μL, 0.1 mmol) was added to a stirred solution of JQ-1 carboxylic acid (10 mg, 0.025 mmol), 2-(2,6-dioxopiperidin-3-yl)-4-(pyrrolidin-3-yloxy)­isoindoline-1,3-dione (8.6 mg, 0.025 mmol), and TBTU (8.4 mg, 0.026 mmol) in DMF (0.5 mL). The reaction was allowed to perform at room temperature overnight. The mixture was diluted with EtOAc, washed with NaHCO_3_ sat. aq., dried over sodium sulfate, filtered, and concentrated to dryness. The resulting material was purified using automated flash chromatography (12 g silica cartridge, DCM/MeOH: 0–10% over 7 column volumes) to provide compound **17** (13.5 mg, 75% yield) as a mixture of diastereoisomers (2/3 ratio).

Major diastereoisomer: ^1^H NMR (400 MHz, DMSO-*d*_6_): δ 11.12 (s, 1H), 7.84 (d, *J* = 7.3 Hz, 1H), 7.60 (d, *J* = 8.6 Hz, 1H), 7.56–7.32 (m, 5H), 5.39–5.30 (m, 1H), 5.14–5.04 (m, 1H), 4.61–4.52 (m, 1H), 4.01 (td, *J* = 9.9, 9.0, 2.7 Hz, 1H), 3.87–3.77 (m, 1H), 3.75–3.56 (m, 2H), 3.53–3.33 (m, 2H), 2.96–2.81 (m, 1H), 2.65–2.51 (m, 5H), 2.42 (s, 3H), 2.40–2.12 (m, 2H), 2.10–1.97 (m, 1H), 1.64 (s, 3H). ^13^C NMR (101 MHz, DMSO-*d*_6_): δ: 173.2, 170.4, 168.8, 167.2, 165.7, 163.5, 155.7, 154.8, 150.3, 137.5, 137.3, 135.6, 134.0, 132.7, 131.2, 130.7 (2C), 130.4, 130.1, 128.9 (2C), 122.0, 117.9, 116.4, 77.5, 54.5, 52.1, 49.2, 44.8, 36.8, 31.8, 31.4, 22.5, 14.5, 13.2, 11.8.

Minor diastereoisomer: ^1^H NMR (400 MHz, DMSO-*d*_6_): δ 11.10 (s, 1H), 7.85 (d, *J* = 7.3 Hz, 1H), 7.64 (d, *J* = 8.6 Hz, 1H), 7.56–7.32 (m, 5H), 5.47–5.40 (m, 1H), 5.14–5.04 (m, 1H), 4.61–4.52 (m, 1H), 4.13 (dd, *J* = 11.9, 4.5 Hz, 1H), 3.95–3.87 (m, 1H), 3.75–3.56 (m, 2H), 3.53–3.33 (m, 2H), 2.96–2.81 (m, 1H), 2.65–2.51 (m, 5H), 2.42 (s, 3H), 2.40–2.12 (m, 2H), 2.10–1.97 (m, 1H), 1.64 (s, 3H). ^13^C NMR (101 MHz, DMSO-*d*_6_): δ: 173.2, 170.4, 168.6, 167.2, 165.7, 163.4, 155.6, 154.8, 150.3, 137.4, 137.2, 135.6, 134.0, 132.6, 131.2, 130.6 (2C), 130.3, 130.1, 129.0 (2C), 121.9, 117.8, 116.4, 78.9, 54.3, 51.6, 49.2, 44.0, 36.9, 31.8, 30.5, 22.5, 14.5, 13.2, 11.8.

HRMS, ESI^+^ *m*/*z*: calcd for C_36_H_32_ClN_7_O_6_S [M + H]+, 726.1896; found, 726.1890.

### Statistics

Statistical analyses were performed in R (v.4.2.2). The goodness of fit for the linear regressions was determined by calculating the coefficient of determination (*R* ^2^) with the R package ggpmisc (v 0.6.0).

## Supplementary Material





## Data Availability

Detailed supplemental data are available free of charge in the Zenodo repository, as part of record 10.5281/zenodo.17708199. The .xlsx file contains SMILES format structures and separation into E3 ligand, linker, and POI ligand substructures for all 3583 analyzed PROTACs and their original literature references (“Overview” sheet), molecular properties calculated from 2D structures (“2D details” sheet, used in Table 1), molecular properties calculated from 3D structures (“3D details” sheet, Figure 1), details on the PROTAC–membrane simulations (‘Membrane simulations’ sheet, Figures 4–6), details on the experimental measurements of PROTAC–cell membrane interactions (“membrane affinity” sheet, Figures 2 and 6), and Caco-2 cell permeabilities (“Caco-2” sheet, Figure 3).

## Notes

Additional figures and tables referred to in the article, NMR spectra, HPLC traces, and reaction schemes of synthesized compounds (PDF)
Molecular formula strings document with structures (SMILES codes), cell membrane binding data for **1**–**17**, and Caco-2 cell permeability data for **1–10** (CSV)

## Abbreviations

COSMO: continuum solvation model
CRBN: cereblon
DMPC: 1,2-dimyristoyl-sn-glycero-3-phosphocholine
IAP: inhibitors of apoptosis proteins
nRotB: number of rotatable bonds
POI: protein-of-interest
PROTAC: proteolysis targeting chimera
SMILES: simplified molecular input line entry system
TPSA: topological surface area
VHL: von Hippel–Lindau
