# Supplementary material for: Conformational Dynamics in the Cell Membrane Interactions of Bispecific Targeted Degrader Therapeutics
Source: J Med Chem. 2025 Dec 10;68(24):25881–98. doi: 10.1021/acs.jmedchem.5c01499 (PMC12750995; doi:10.1021/acs.jmedchem.5c01499)

Supporting Information

Conformational dynamics in the cell membrane interactions of bispecific targeted degrader therapeutics

Emma Inganäs[1], Hanna Lavén[1], Rémi Caraballo[2], Fredrik Klingegård[2], MyLan Eklund[1], Linda Andersson[3], Constanze Hilgendorf[3], Pär Matsson*[1,4]

[1]Unit for Pharmacokinetics and Drug Metabolism, Department of Pharmacology, Sahlgrenska Academy, University of Gothenburg, 405 30 Gothenburg, Sweden
[2]SciLifeLab Drug Discovery and Development, Medicinal Chemistry Hit2Lead, Karolinska Institute, 171 65 Solna, Sweden
[3]DMPK, Early Research and Development, Cardiovascular, Renal and Metabolism, BioPharmaceuticals R&D, AstraZeneca, 431 53 Gothenburg, Sweden
[4]SciLifeLab Gothenburg, University of Gothenburg, 405 30 Gothenburg, Sweden

*Email: par.matsson@gu.se

Table of Contents

Supporting Tables

Table S1 .... S3
Table S2 .... S3
Table S3 .... S4
Table S4 .... S4

Supporting Figures

Figure S1 .... S5
Figure S2 .... S6
Figure S3 .... S7
Figure S4 .... S7
Figure S5 .... S8
Figure S6 .... S8
Figure S7 .... S9
Figure S8 .... S10
Figure S9 .... S11

NMR spectra

1H-, 13C- and HSQC-NMR spectra of compound **11** S11
1H- and 13C-NMR spectra of compound **12** S13
1H- and 13C-NMR spectra of compound **13** S14
1H- and 13C-NMR spectra of compound **14** S15
1H- and 13C-NMR spectra of compound **15** S16
1H- and 13C-NMR spectra of compound **16** S17
1H-NMR spectra of compound **S-9** S18
1H- and 13C-NMR spectra of compound **17** S19

HPLC traces

HPLC traces of compound **11** S20
HPLC traces of compound **12** S21
HPLC traces of compound **13** S22
HPLC traces of compound **14** S23
HPLC traces of compound **15** S24
HPLC traces of compound **16** S25
HPLC traces of compound **17** S26

Reaction schemes

Reaction schemes for compounds **11-17** S27

Supporting Tables

Table S1. Summary of molecular descriptors (median (min-max range), n = 77) of full PROTAC molecules, and for the separated E3 ligand, linker and POI ligand domains for PROTACs reported to bind to other E3 ligases than CRBN and VHL.

**PROTACs binding to other E3 ligases**				
	**Full molecule**	**E3 ligand**	**Linker**	**POI ligand**
**n types**	77	32	59	35
**MW[a] [Da]**	1051 (774-1514)	482 (271-664)	190 (44-440)	394 (281-860)
**cLogP[b]**	7.5 (2.1-14)	2.5 (0.5-7.5)	0 (-1.3-3.9)	3.8 (1.3-8.6)
**TPSA[c] [Å^2]**	209 (130-281)	92 (26-138)	45 (0-103)	68 (42-116)
**HBD[d]**	5 (1-7)	3 (0-4)	0 (0-2)	1 (0-2)
**HBA[e]**	14 (9-20)	5 (2-9)	4 (0-10)	6 (3-9)
**nRotB[f]**	25 (14-36)	8 (1-11)	10 (1-21)	5 (1-13)

[a] molecular weight; [b] calculated LogP; [c] topological surface area; [d] hydrogen bond donors;
[e] hydrogen bond acceptors; [f] number of rotatable bonds

Table S2. Summary statistics of conformation-dependent molecular properties (n = 911 PROTACs). Values are presented as medians (min-max ranges).

	**All PROTACs**		
**n conformations in chloroform**	25858		
**n conformations in water**	43521		
	**CRBN[d]-binding**	**VHL[e]-binding**	**Other**
**n PROTACs**	490	398	23
**TPSA$_{\text{full molecule}}$[a]**	221 (96-793) Å^2	219 (124-353) Å^2	225 (183-257) Å^2
**TPSA$_{\text{E3 ligand}}$[a]**	96 (58-175) Å^2	112 (95-152) Å^2	103 (62-120) Å^2
**3D SA PSA[b] chloroform**	204 (50-536) Å^2	150 (41-319) Å^2	140 (65-233) Å^2
**3D SA PSA[b] water**	257 (76-716) Å^2	193 (44-469) Å^2	180 (81-298) Å^2
**% 3D SA PSA[b] of TSA[c] in chloroform**	20% (5%-44%)	13% (4%-28%)	12% (6%-18%)
**% 3D SA PSA[b] of TSA[c] in water**	25% (7%-50%)	17% (6%-38%)	15% (10%-24%)
**Difference water-chloroform**	51 (-64-237) Å^2	41 (-22-196) Å^2	35 (-21-114) Å^2
**Maximum shielded 3D SA PSA[b]**	117 (6-300) Å^2	97 (25-248) Å^2	92 (48-190) Å^2
**Normalized shielded 3D SA PSA[b]**	40% (2%-80%)	43% (14%-76%)	42% (24%-70%)

[a] topological surface area; [b] solvent accessible polar surface area; [c] solvent accessible total surface area;
[d] cereblon; [e] von Hippel-Lindau

Table S3. Summary statistics of the ability of PROTAC molecules (n = 211) to hide polar surface area. The difference in PSA from membrane core to water has been calculated from the Boltzmann weighted conformer ensembles.

Group	% of molecules	Difference in PSA from membrane core to water
PSA and TSA higher in membrane core than in water	41.6%	0-82 $Å^2$
PSA higher and TSA lower in membrane core than in water	4.3%	0-7 Å
PSA lower and TSA higher in membrane core than in water	27.1%	-36-0 $Å^2$
PSA and TSA lower in membrane core than in water	27.1%	-102-0 $Å^2$
> 10 $Å^2$ higher PSA in membrane core than in water	11.1%	
0-10 $Å^2$ higher PSA in membrane core than in water	34.8%	
0-10 $Å^2$ lower PSA in membrane core than in water	36.8%	
> 10 $Å^2$ lower PSA in membrane core than in water	17.4%	

Table S4. Mass transitions, cone voltages, collision energies.

Compound	ESI mode	Charge	Parent	Daughter	Cone (V)	Collision (eV)
1	ESI+	+2	560.9951	318.2010	34	16
2	ESI+	+2	512.9183	318.0852	16	28
3	ESI+	+2	499.7578	127.1340	22	22
4	ESI+	+2	557.4903	318.0979	16	16
5	ESI+	+2	469.4172	358.0882	16	16
6	ESI+	+2	501.9786	188.1866	16	28
7	ESI+	+2	390.8361	309.1414	16	22
8	ESI+	+1	841.2766	383.0578	10	46
9	ESI+	+1	785.2534	383.0003	10	40
10	ESI+	+2	392.8116	309.1384	28	22
11	ESI+	+2	457.1584	318.1621	22	16
12	ESI+	+1	781.4137	383.1128	10	34
13	ESI+	+1	713.3591	383.0981	22	34
14	ESI+	+1	845.3931	383.1785	16	40
15	ESI+	+1	725.3309	383.1176	22	34
16	ESI+	+1	801.3659	341.1367	28	46
17	ESI+	+1	726.3359	383.1140	16	34
Lopinavir	ESI+	+1	629.4825	155.1768	40	46
Atorvastatin	ESI+	+1	559.3015	440.1533	34	22
Verapamil	ESI+	+1	455.2701	165.0336	70	28

Supporting Figures

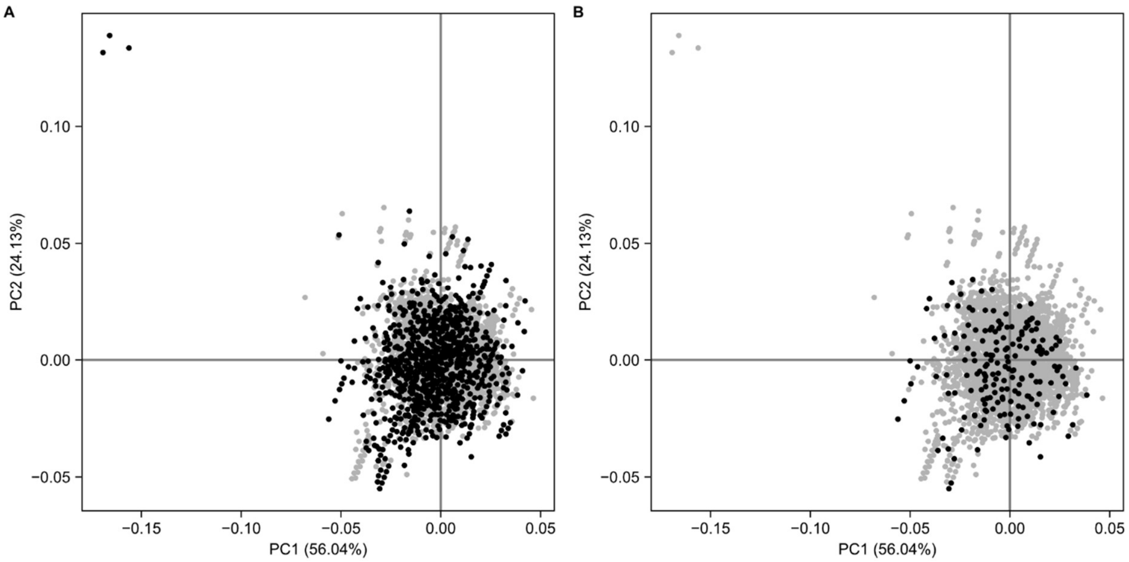

Figure S1. Principal Component Analysis (PCA) of all PROTACs included in the dataset (n = 3576). A) PROTACs selected in 3D analysis highlighted in black (n = 911). The three PROTACs located in the upper left corner are peptide-based PROTACs with substantially larger molecular weight and containing many more hydrogen bonding functions than all other PROTACs in the database. B) PROTACs simulated with membrane highlighted in black (n = 210).

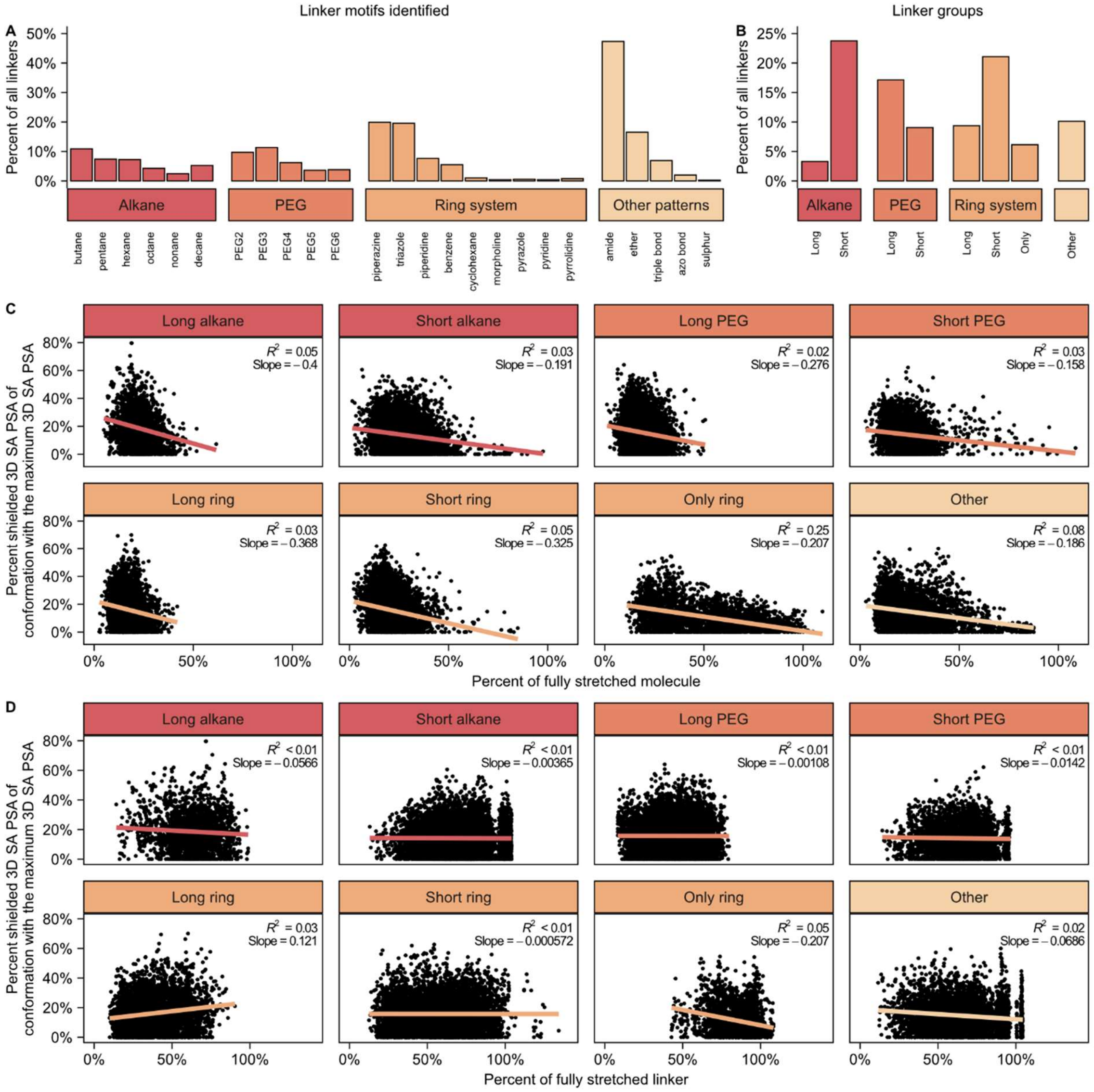

Figure S2. Linker characteristics and origins and impact of PROTAC folding. A) Linker patterns identified in the dataset (n = 3576). One linker fragment can contain several linker patterns, or none, leading to cumulative percentages that can exceed 100%. B) Linker groups based on chain length and chemistry; long/short alkane (≥9/<9 carbons), long/short PEG (≥3/<3 PEG monomers), long/short ring systems (any linker containing a ring in combination with a long/short alkane or PEG segment), only ring systems (no alkane or PEG segment attached to ring) and other (linkers that fit to several groups or to none). Each linker (n = 3576) was only assigned to a single group. C) Percent shielding of each type of linker group indicates that more shielding occurs in molecules which are folded to a higher degree (n = 911). D) No correlation seen between shielding of PSA and linker folding (n = 911).

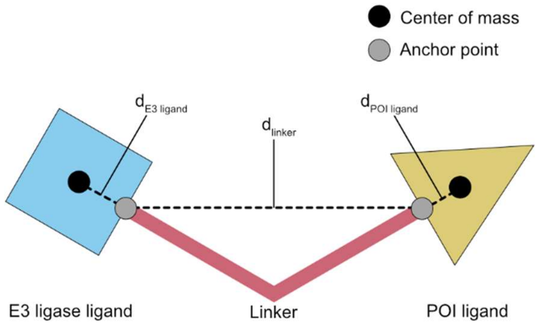

Figure S3. Graphical illustration of how the linker and molecule contraction term were calculated. The sum of distances *E3 ligase center of mass to E3 ligase ligand anchor point* ($d_{E3\ ligand}$), *E3 ligase ligand anchor point to POI ligand anchor point* (d_{linker}) and *POI ligand anchor point to POI ligand center of mass* ($d_{POI\ ligand}$) was calculated for 2D and 3D conformations. Molecule contraction was calculated using Equation 2 and linker contraction was calculated using Equation 3 (see Methods section in the main article).

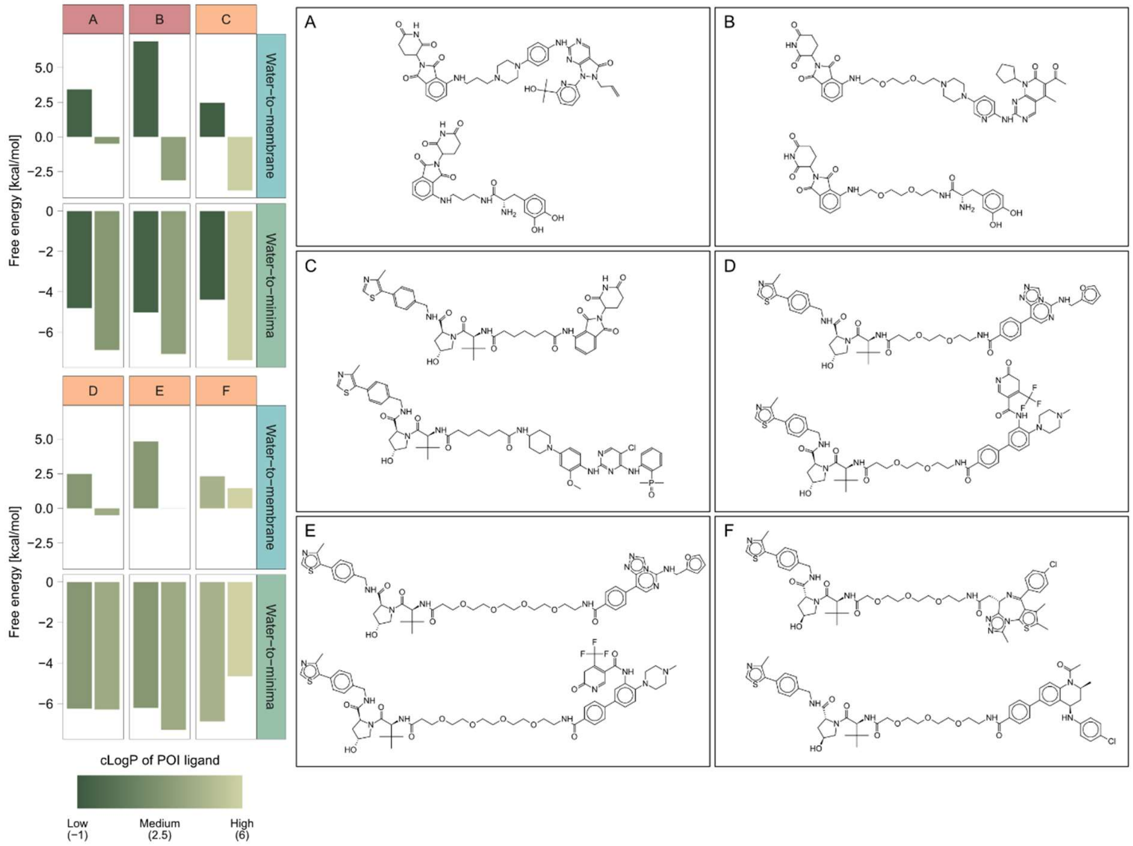

Figure S4. Influence of POI ligand on energy barriers in linker- and E3 ligand-matched PROTACs (left). Darker color represents a lower cLogP and a lighter color represents a higher cLogP of the POI ligand. Molecular structures of the compounds to the right.

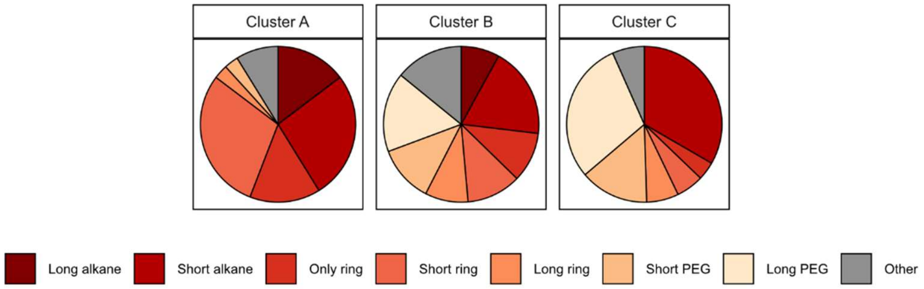

Figure S5. Cluster characteristics (n = 210 PROTACs). Long PEG-based linkers are more common in cluster C, which has a higher energy barrier at the bilayer center. In contrast, more rigid and alkane-based linkers are more common in cluster A, which exhibits a deeper energy minima.

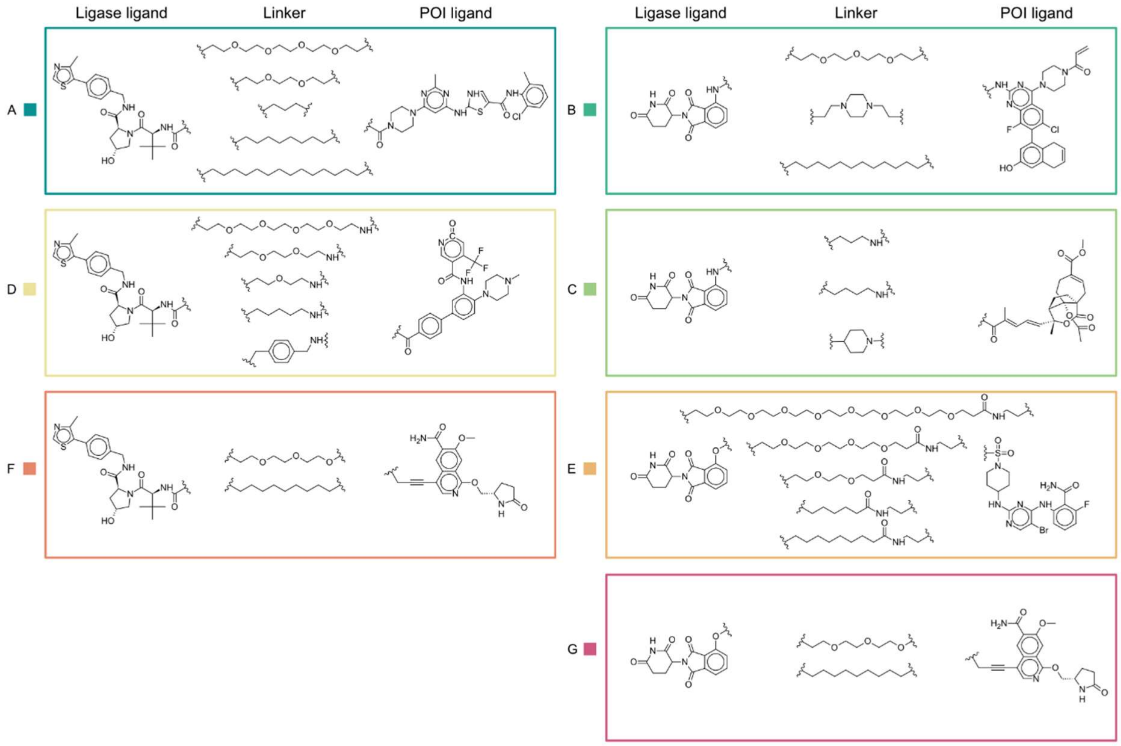

Figure S6. Molecular structures of PROTAC linker series A-G included in Figure 4G.

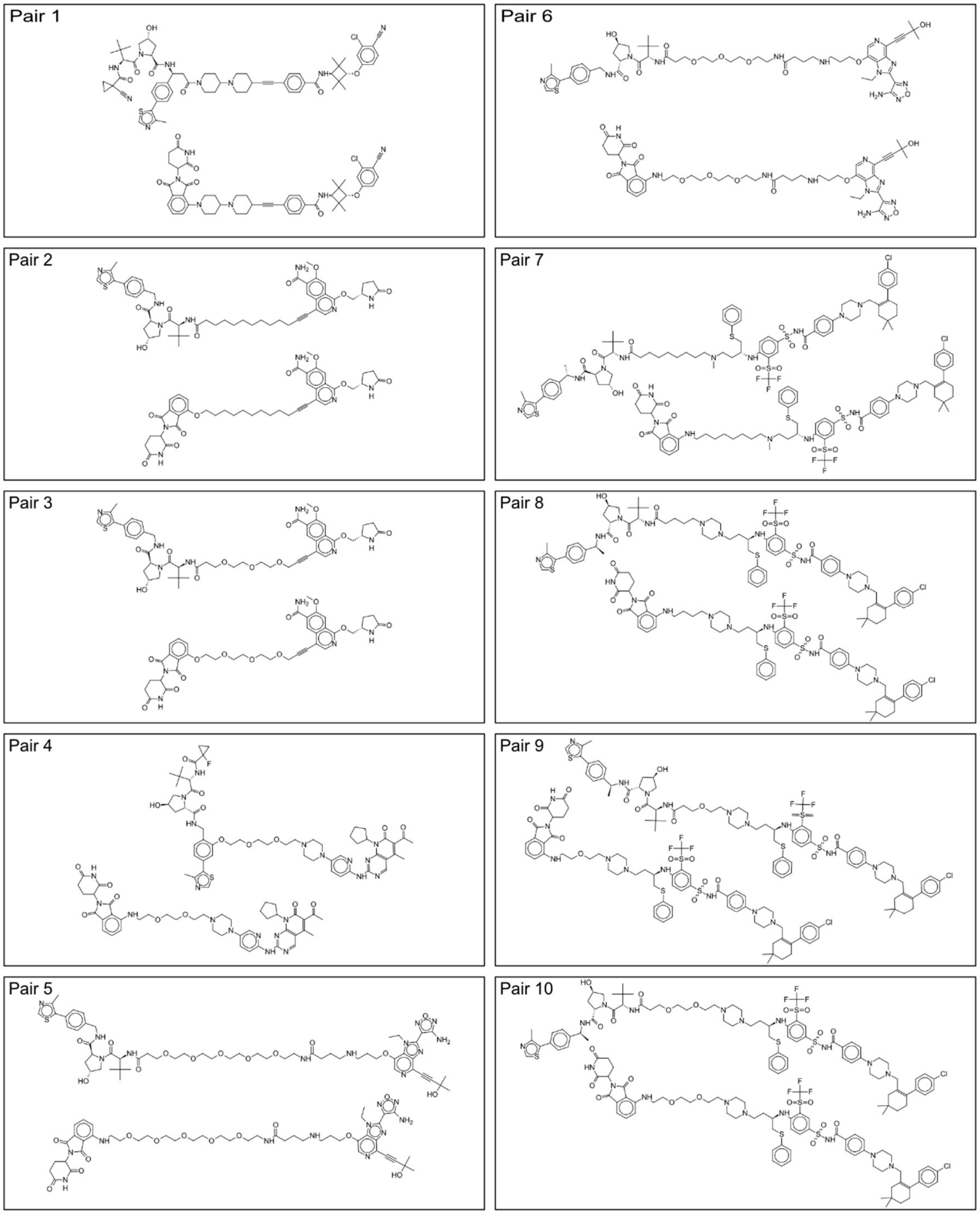

Figure S7. Molecular structures of PROTAC matched pairs included in Figure 4D.

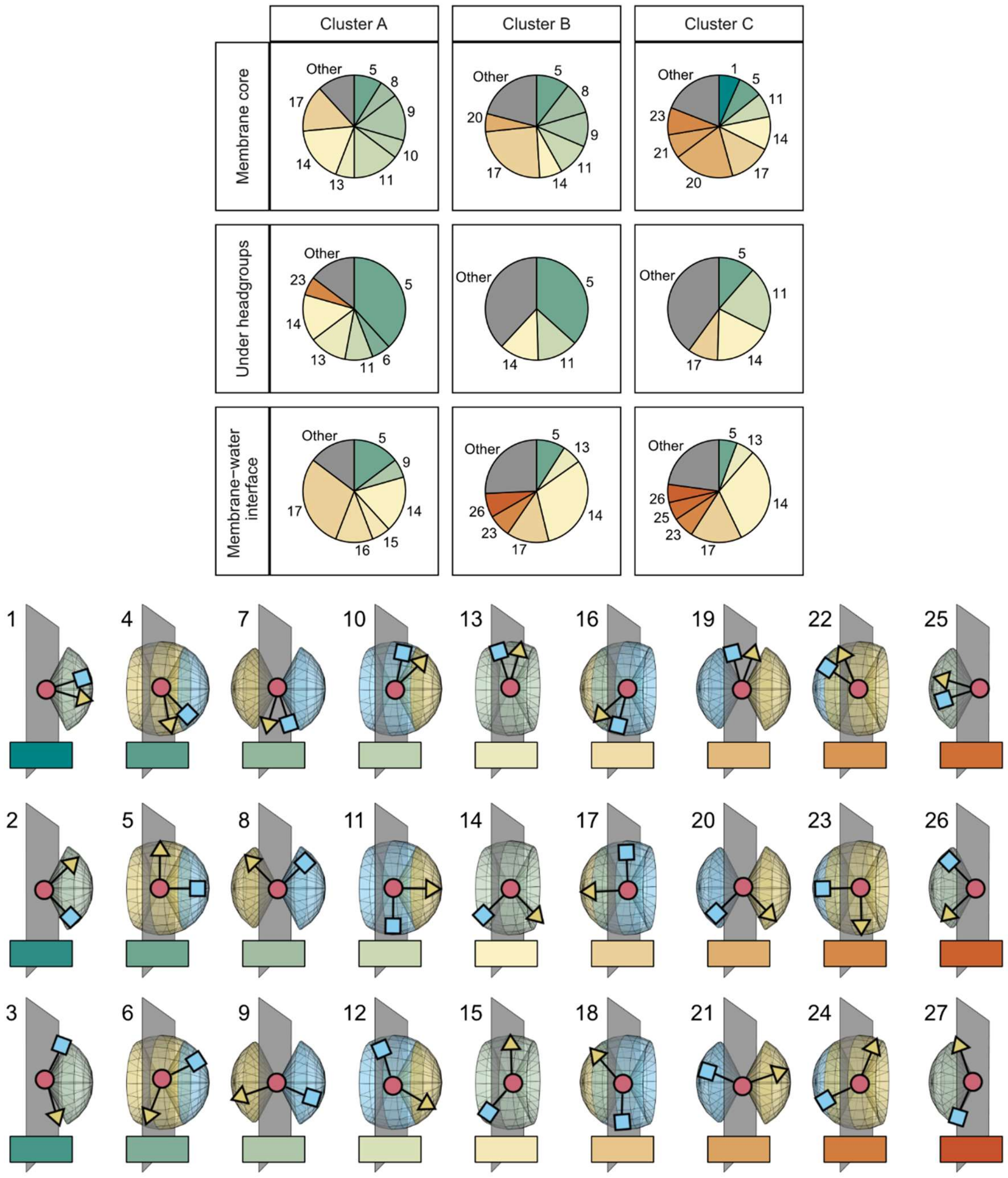

Figure S8. PROTAC angles in the different clusters (n = 210 PROTACs). All orientation groups that more than 5% of molecules in each cluster belong to are indicated by a number, and those which contain less than 5% of molecules in each cluster are grouped together and marked Other. In Cluster A, with molecules with a deeper energy minima, a slight preference for extended, more linear conformations are seen, with the POI ligand pointing in towards the core and ligase ligand pointing towards the water. In Cluster C, with molecules with high energy barriers at the bilayer center, the ligase ligand more often points in towards the core and the POI ligand points out from the core.

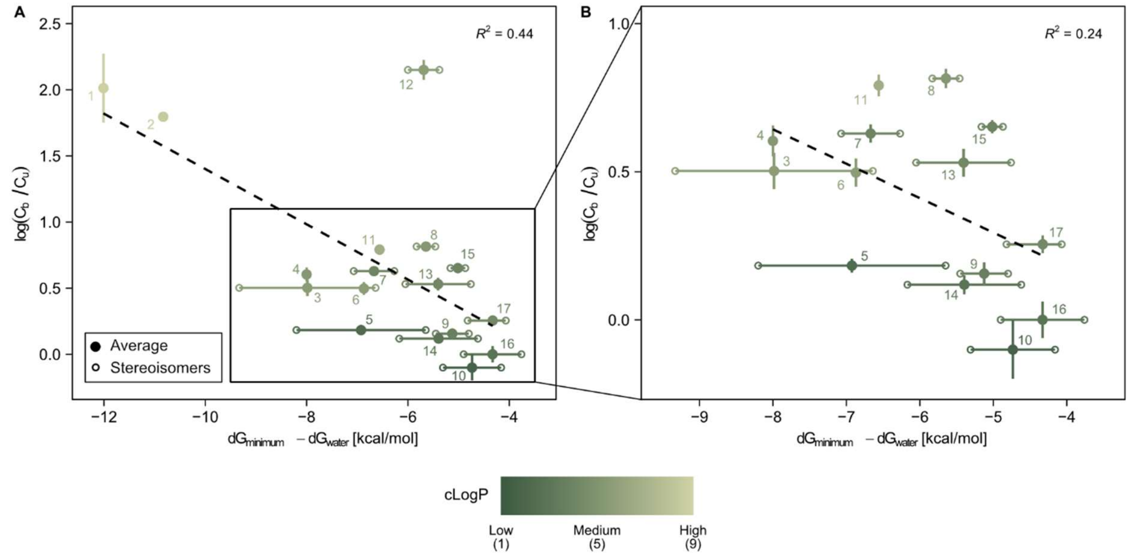

Figure S9. Correlation between energy minimum and experimental membrane binding, expressed as the logarithm of bound-to-unbound concentration ratios (mean ± SD, n = 3). Full circles show the average of a 1:1 racemic mixture and open circles show values for the individual stereoisomers. Panel A) shows the correlation in the full experimental dataset and panel B) the correlation when excluding compounds 1, 2 and 12, confirming the general trend of increasing homogenate binding with deeper energy minima also when excluding potential high-leverage data points. The fact that PROTACs **1** and **2** represent different structural series further supports the general trend.

NMR spectra

1H-, 13C- and HSQC-NMR spectra of compound **11**

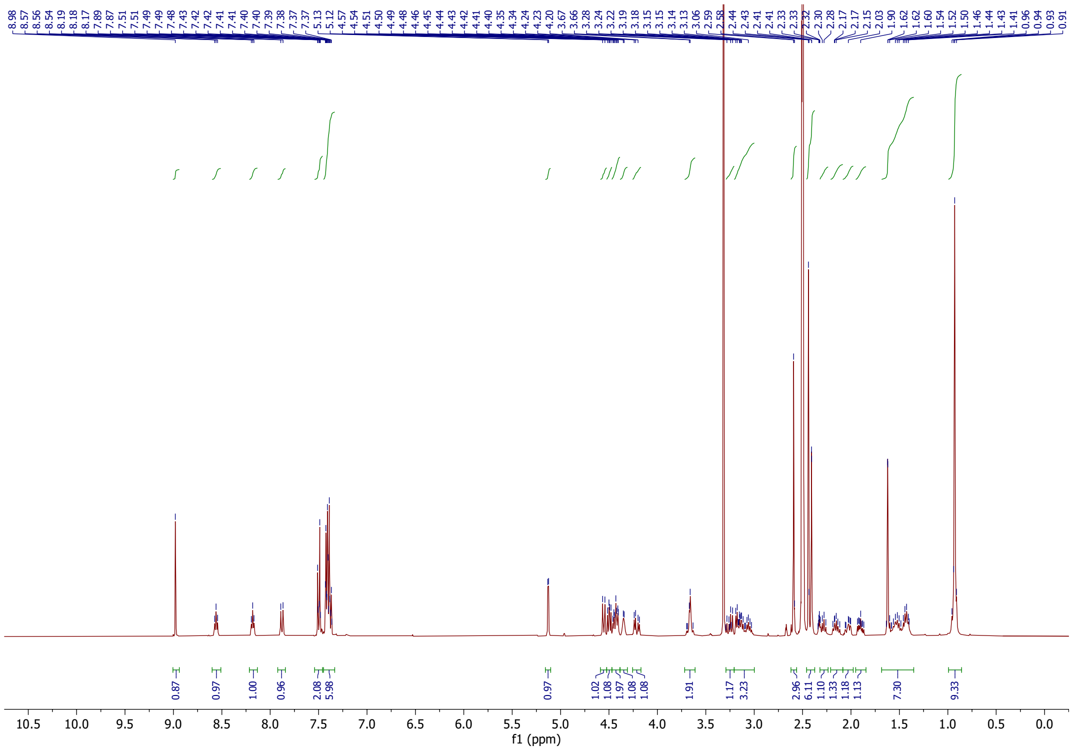

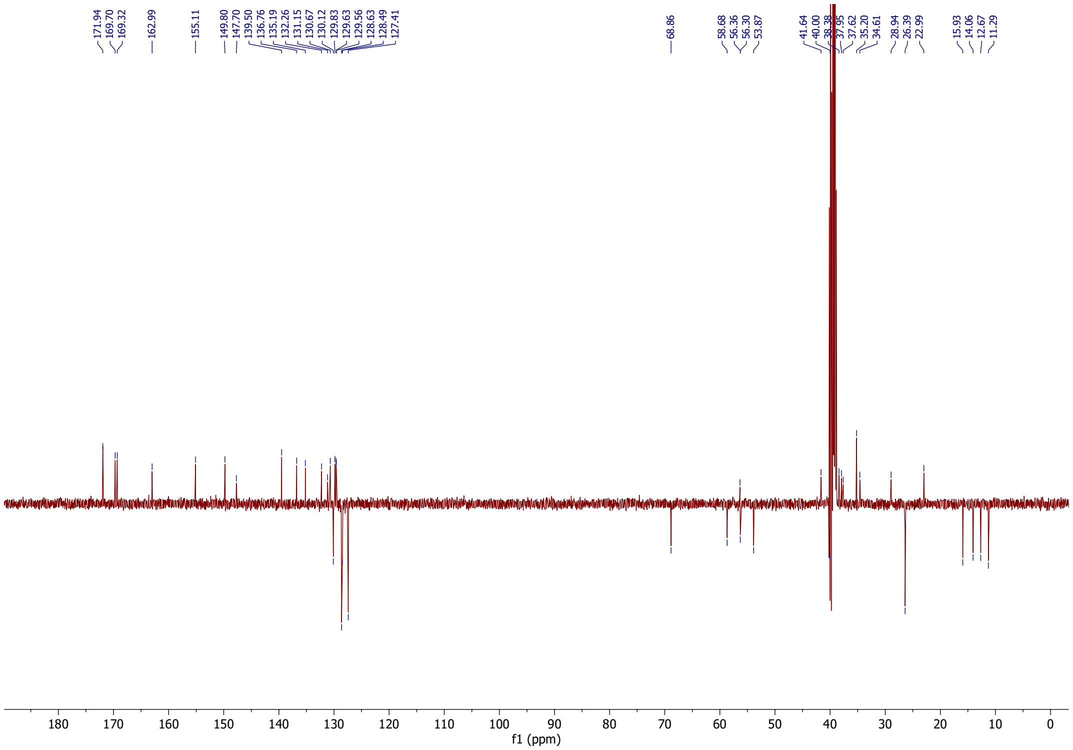

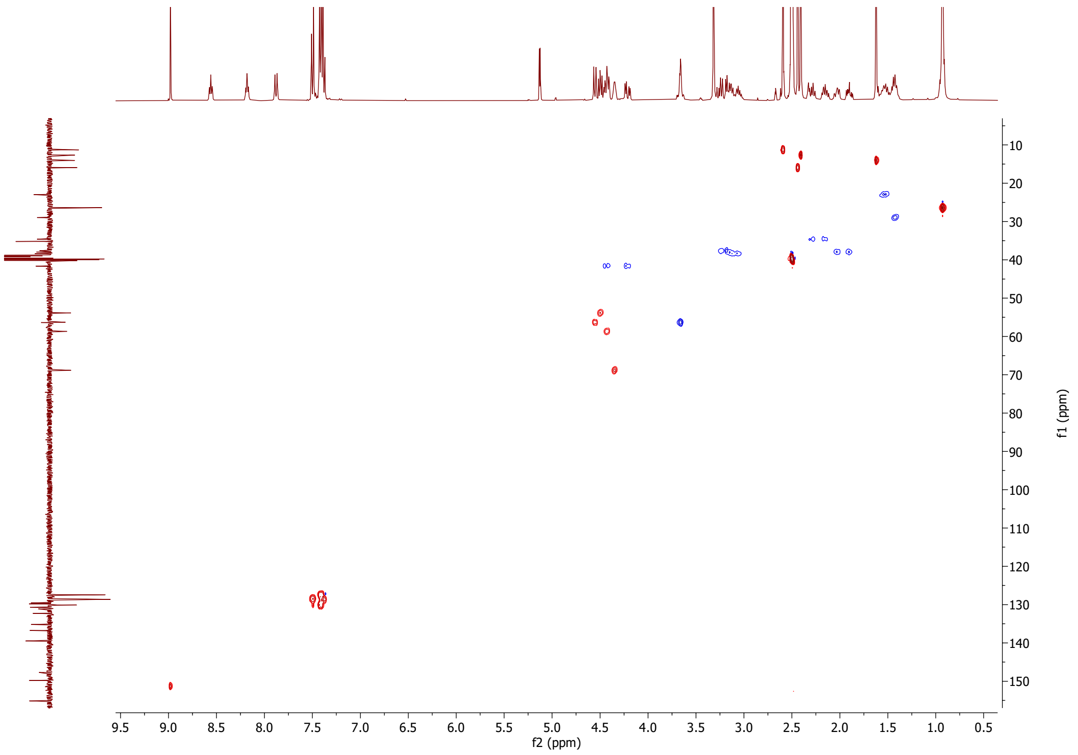

f1 (ppm)
f2 (ppm)

1H- and 13C-NMR spectra of compound **12**

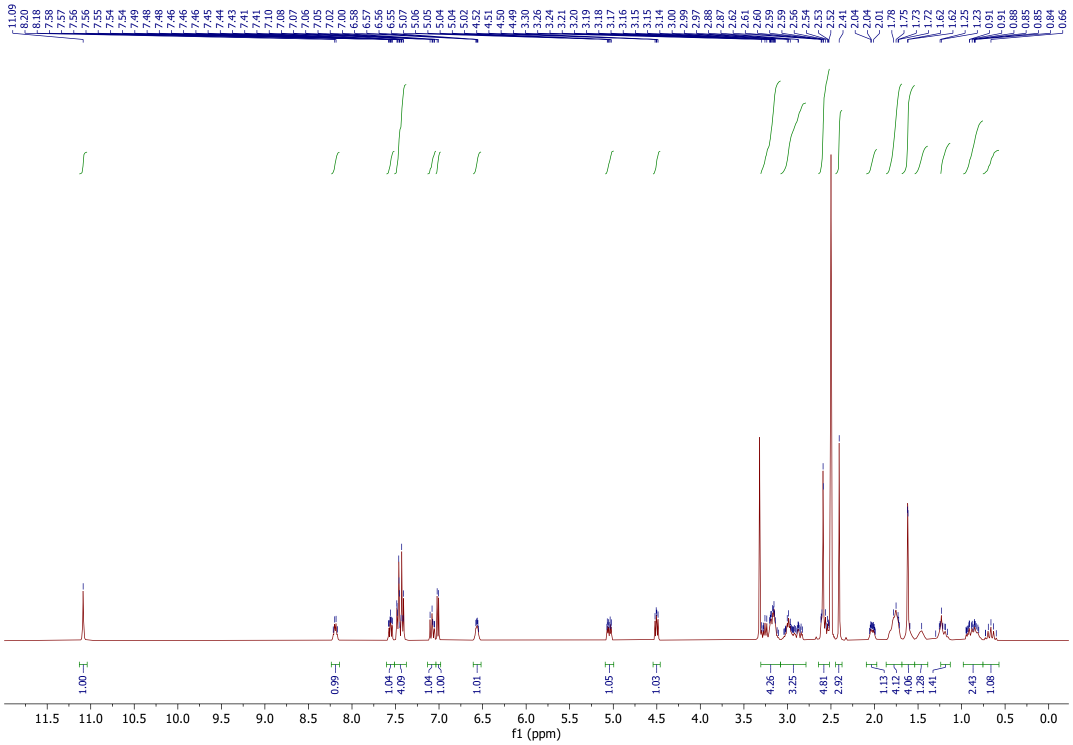

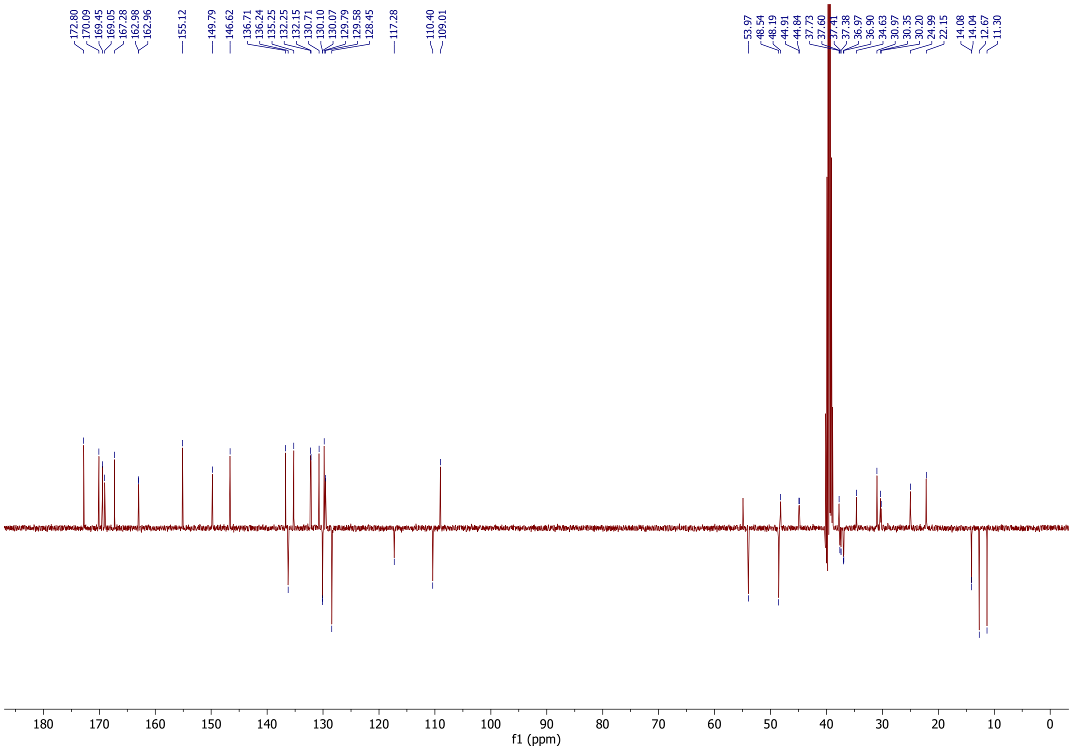

1H- and 13C-NMR spectra of compound **13**

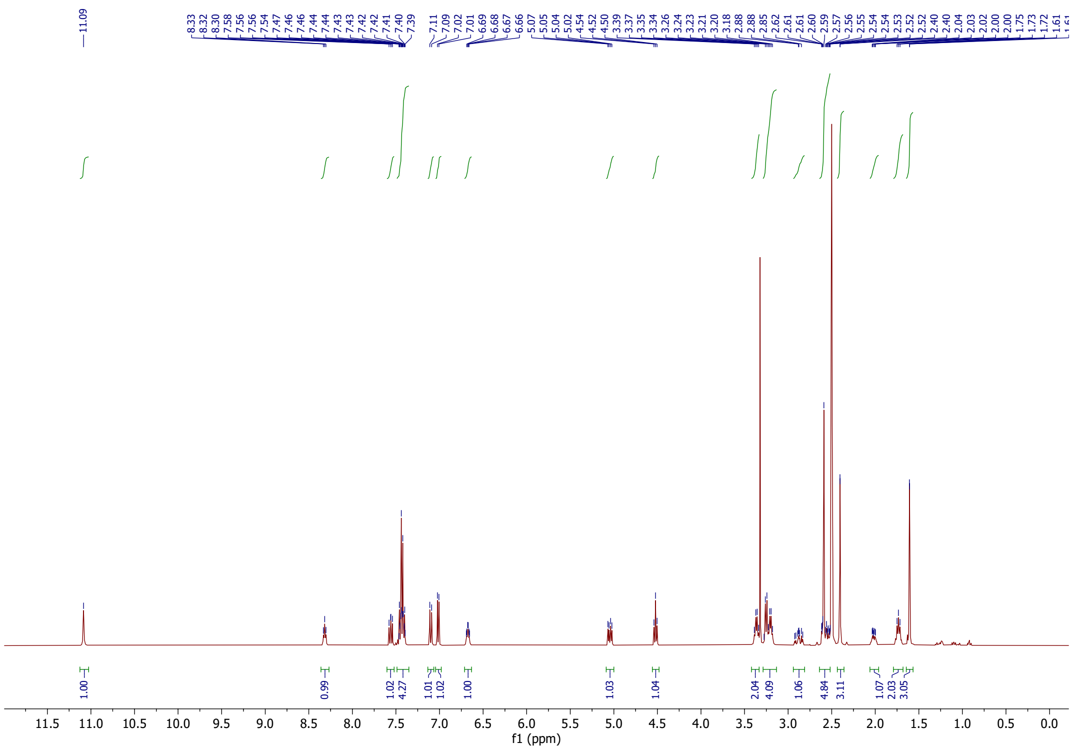

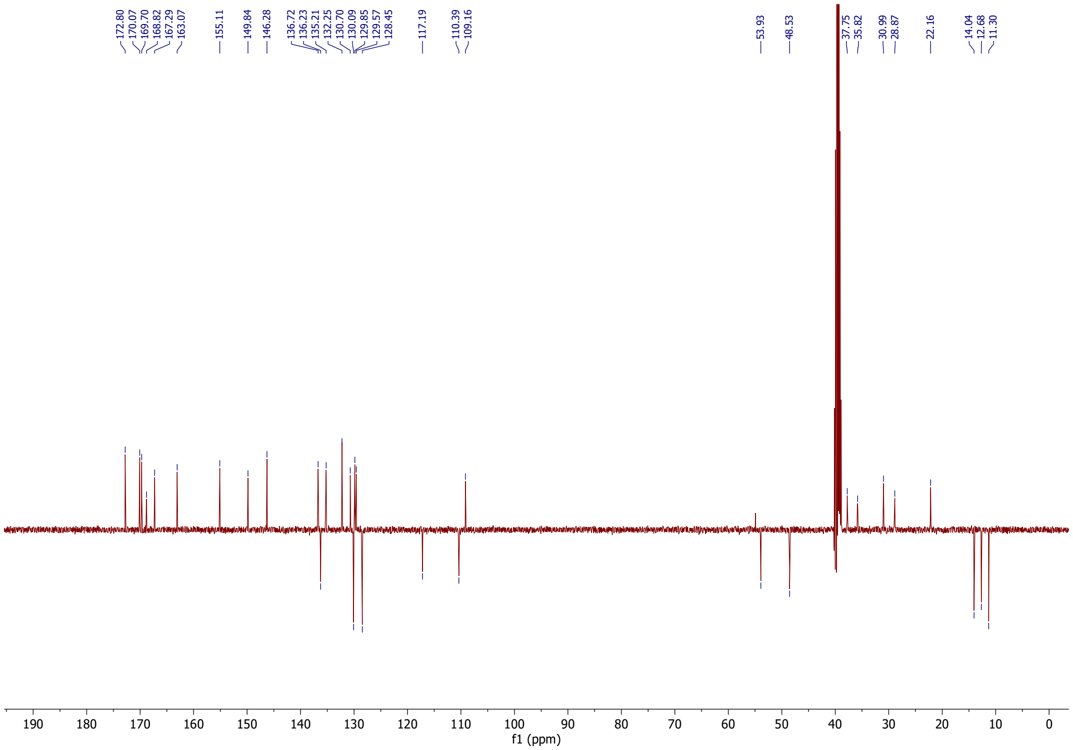

1H- and 13C-NMR spectra of compound **14**

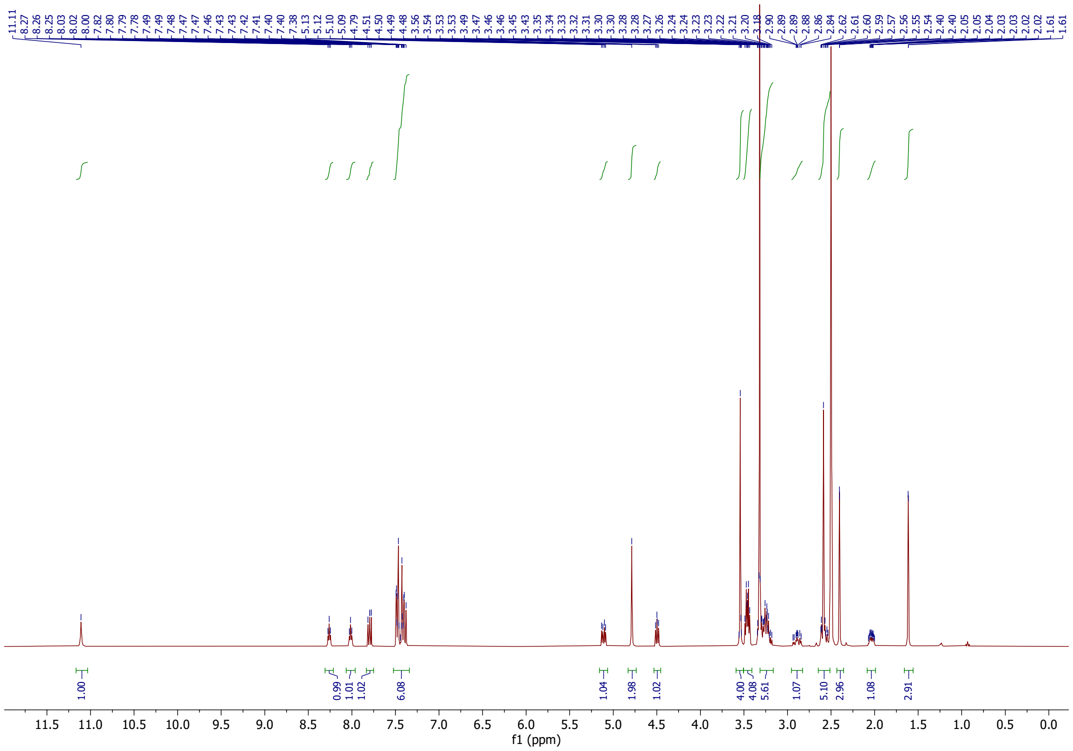

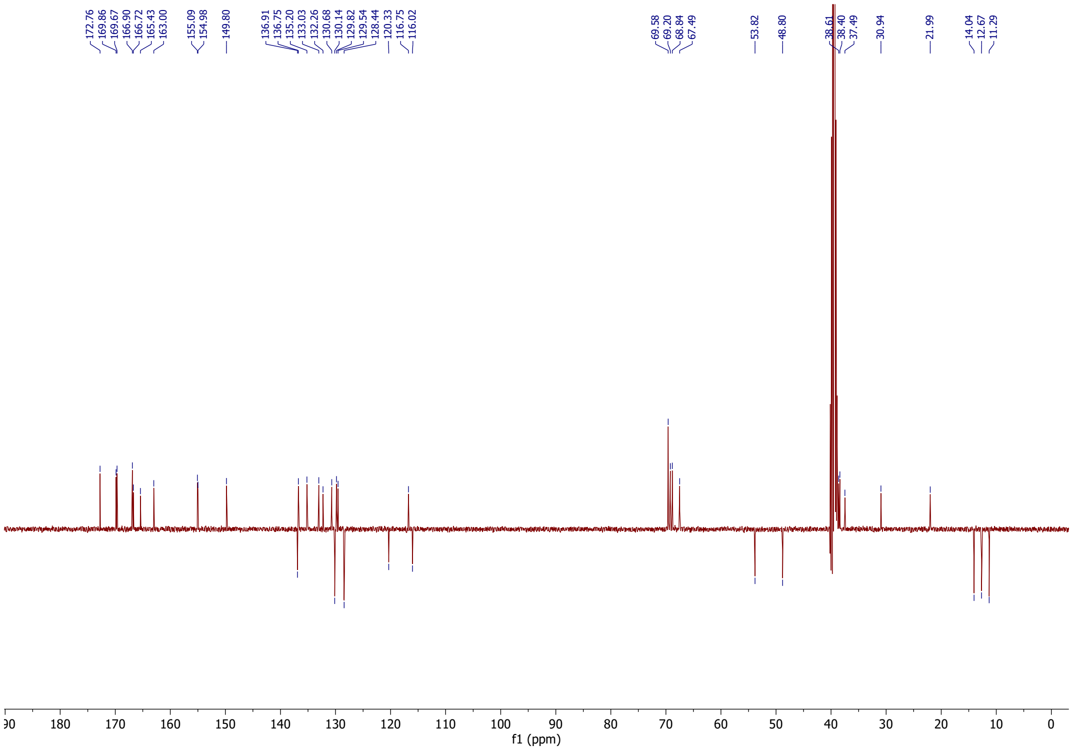

1H- and 13C-NMR spectra of compound **15**

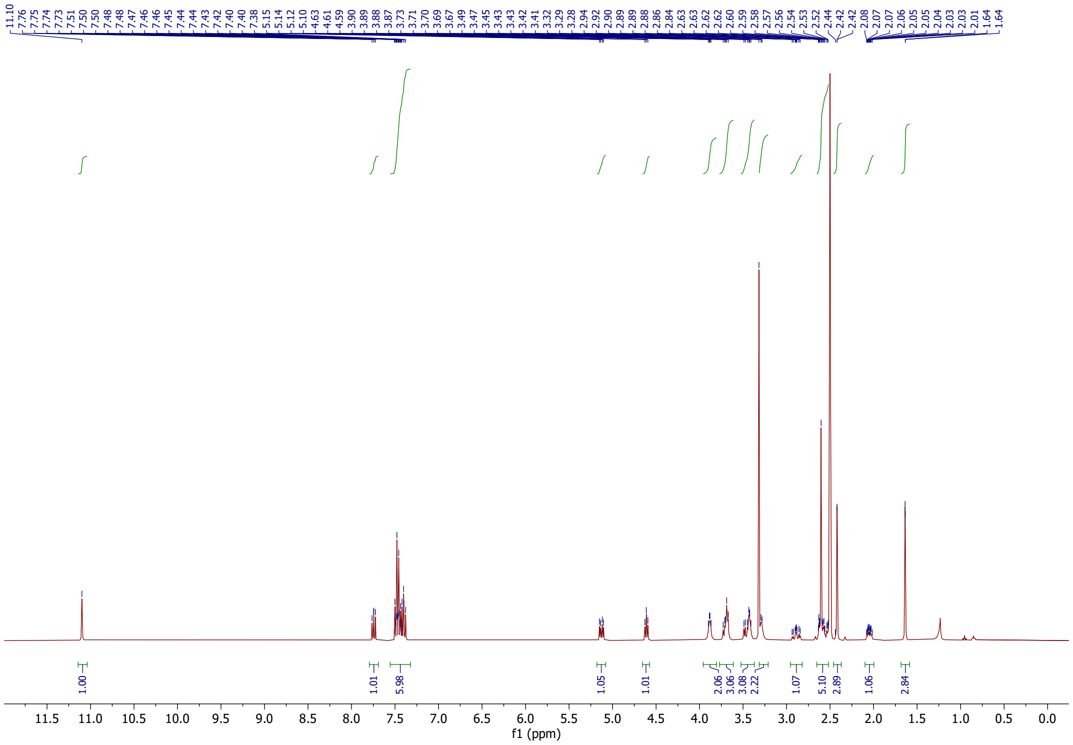

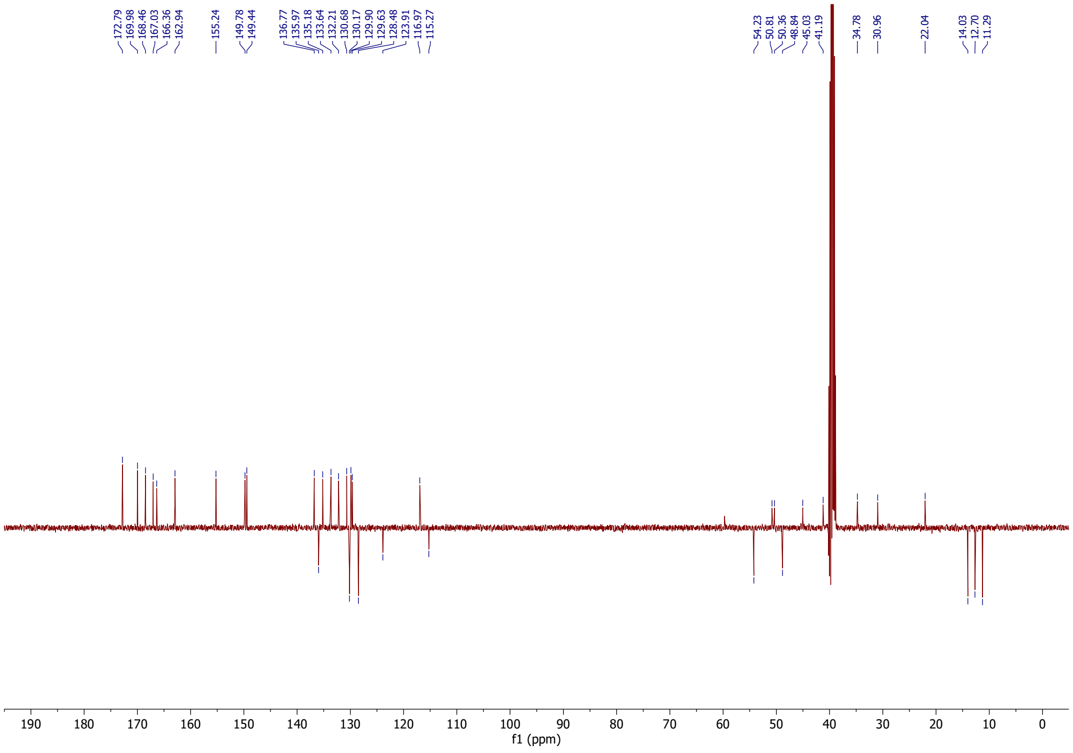

1H- and 13C-NMR spectra of compound **16**

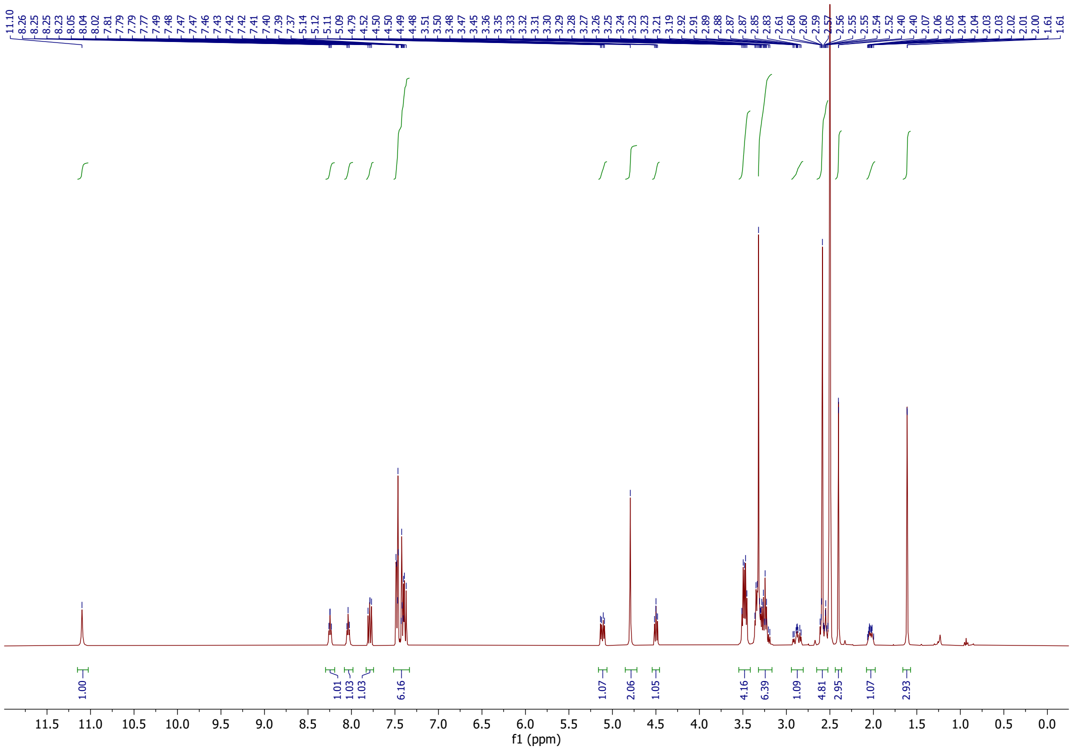

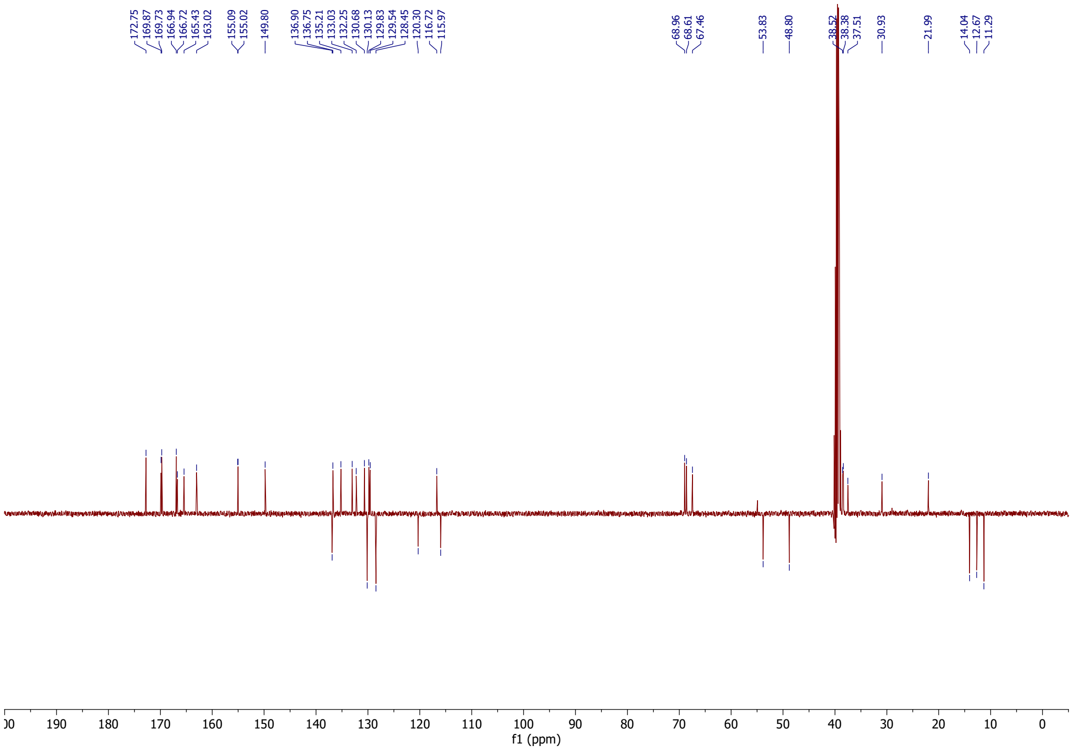

1H-NMR spectra of compound **S-9**

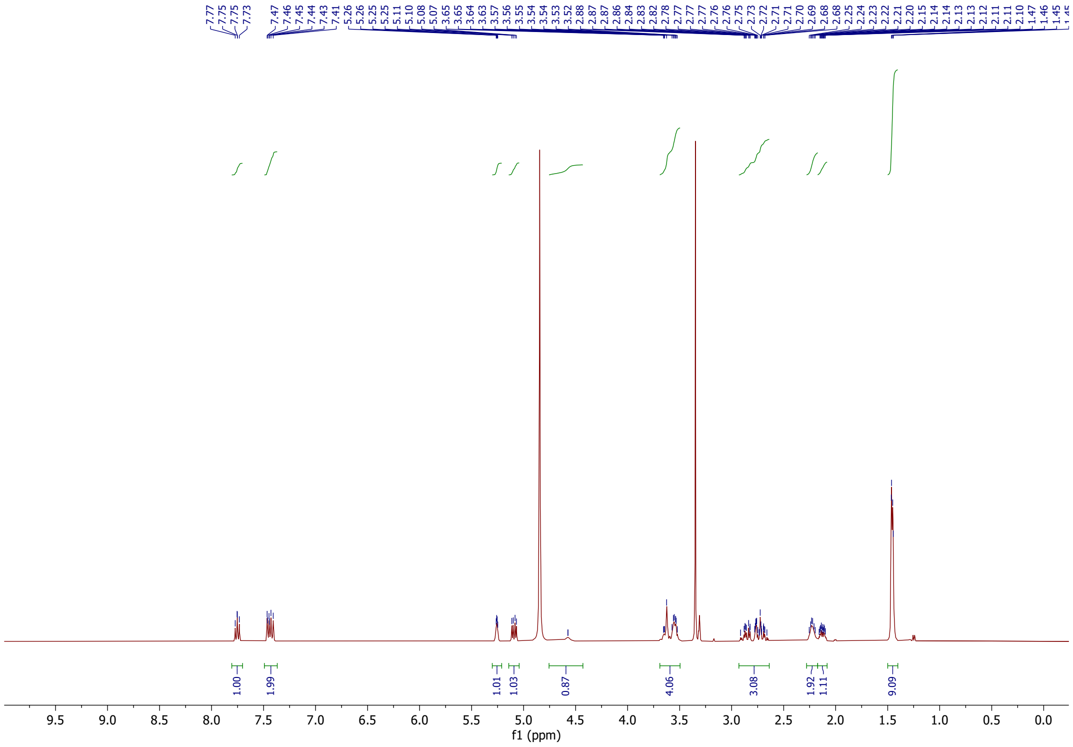

1H- and 13C-NMR spectra of compound **17**

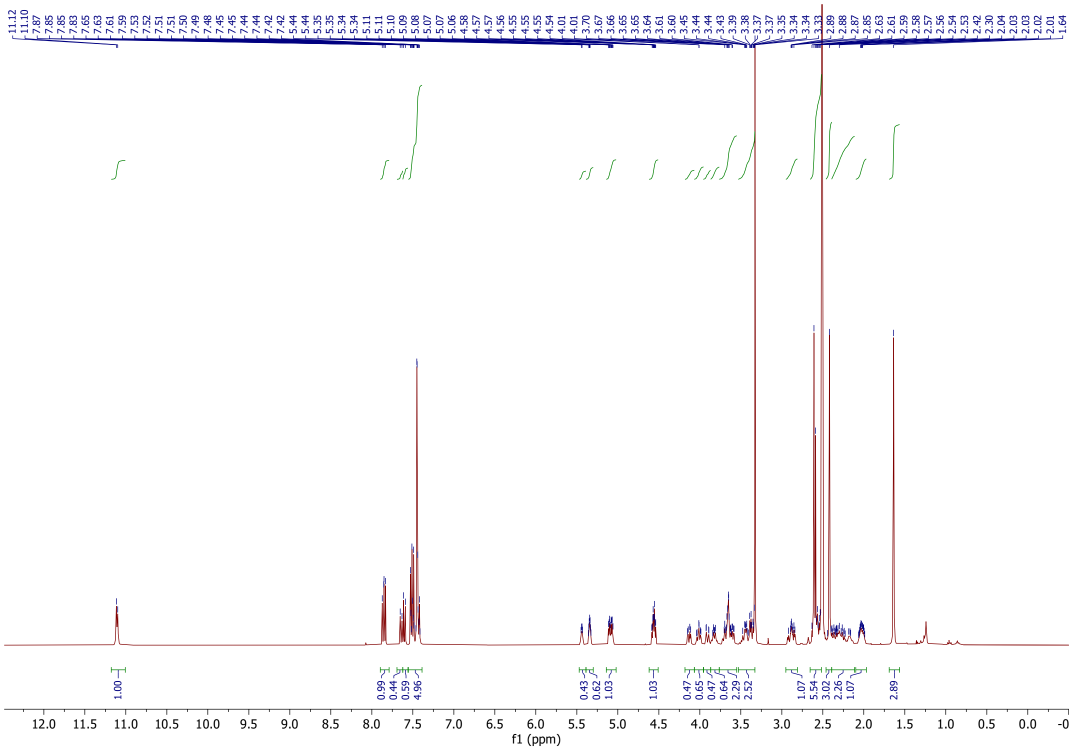

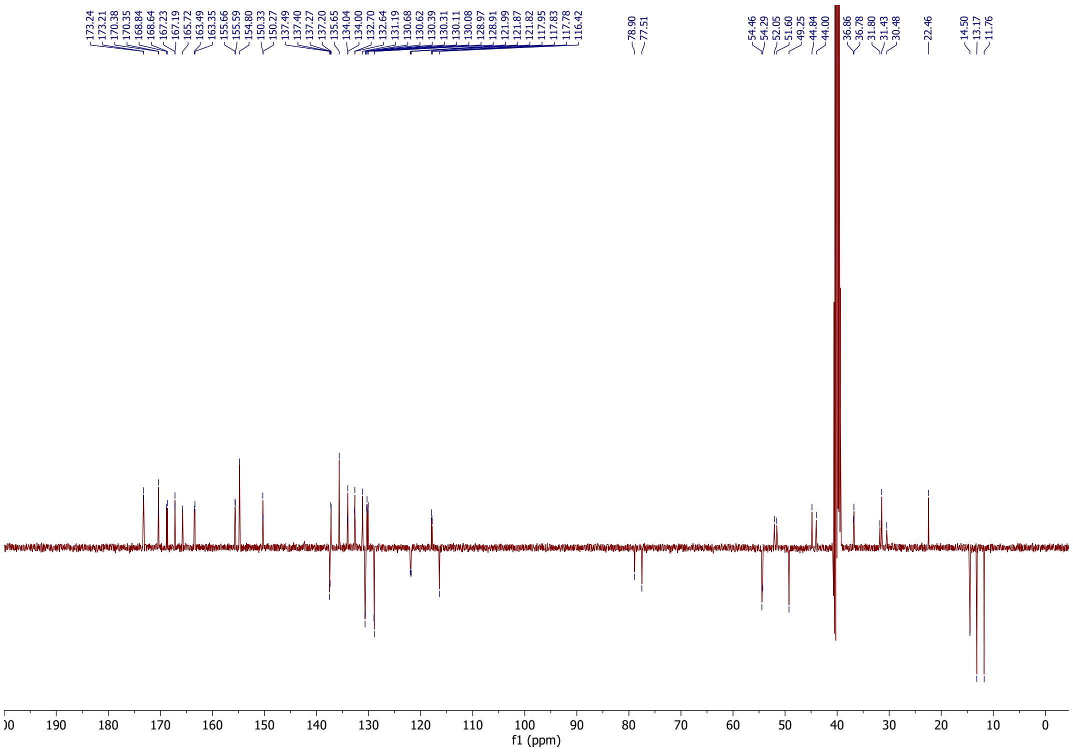

HPLC traces

HPLC traces of compound **11**

Compound 11

```
Method Info     : ACE C8, 50x3mm, 3µ, 10-97% MeCN, 3min; 1ml/min, A: water 0.1% TFA B MeCN
Sample Info     : Walkup method: 'A1097-3'
                  Target:
```

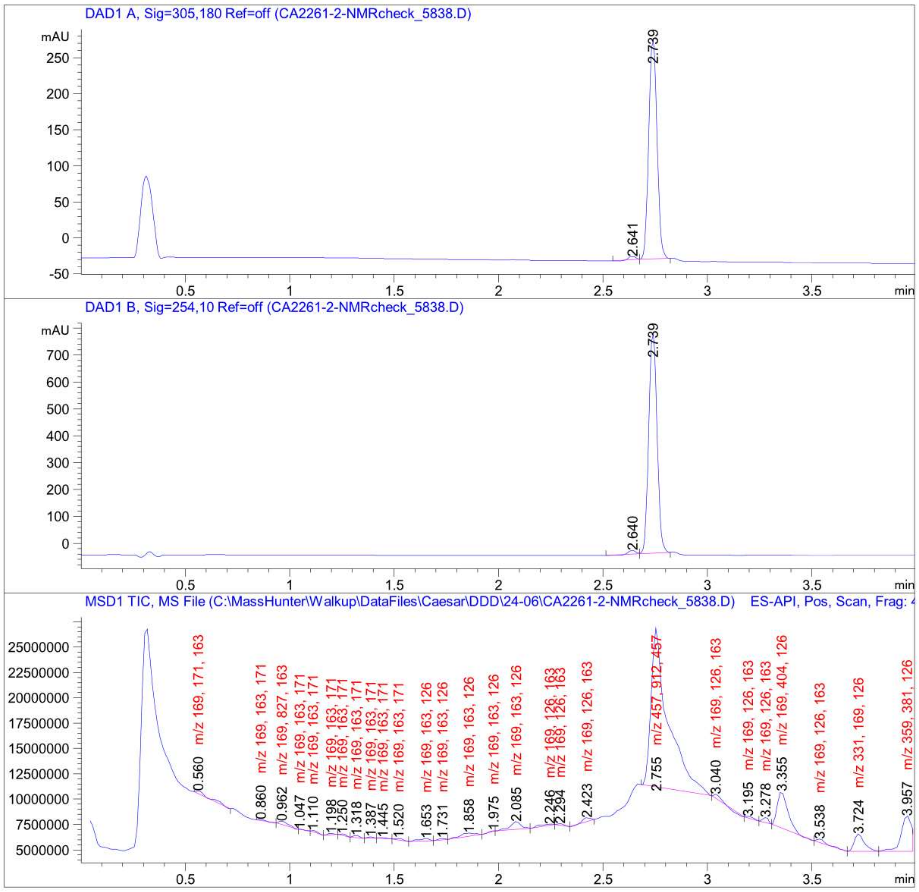

Signal 1: DAD1 A, Sig=305,180 Ref=off

Peak #	RetTime [min]	Type	Width [min]	Area [mAU*s]	Height [mAU]	Area %
1	2.641	BB	0.0364	9.38998	4.48823	1.0993
2	2.739	BB	0.0445	844.76526	303.36795	98.9007

Totals : 854.15524 307.85618

Signal 2: DAD1 B, Sig=254,10 Ref=off

Peak #	RetTime [min]	Type	Width [min]	Area [mAU*s]	Height [mAU]	Area %
1	2.640	BB	0.0338	23.42569	12.55861	1.0187
2	2.739	BB	0.0445	2276.09741	815.85846	98.9813

Totals : 2299.52310 828.41707

HPLC traces of compound **12**

Compound 12

```
Method Info     : ACE C8, 50x3mm, 3µ, 10-97% MeCN, 3min; 1ml/min, A: 0.1% TFA, B:MeCN.

Sample Info     : Walkup method: 'A1097-3'
                  Target:
```

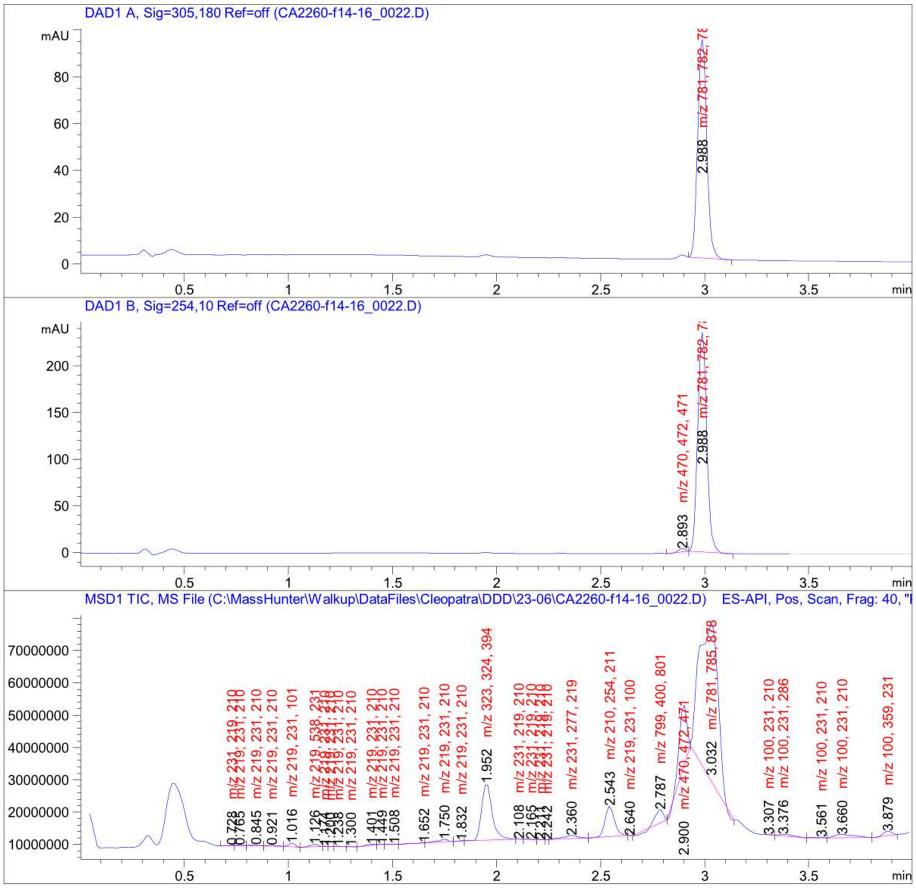

Signal 1: DAD1 A, Sig=305,180 Ref=off

Peak #	RetTime [min]	Type	Width [min]	Area [mAU*s]	Height [mAU]	Area %
1	2.988	BB	0.0473	283.81290	93.70444	100.0000
Totals :				283.81290	93.70444	

Signal 2: DAD1 B, Sig=254,10 Ref=off

Peak #	RetTime [min]	Type	Width [min]	Area [mAU*s]	Height [mAU]	Area %
1	2.893	BB	0.0379	7.48719	3.36876	1.0406
2	2.988	BB	0.0473	712.02216	235.57503	98.9594
Totals :				719.50935	238.94379	

HPLC traces of compound **13**

Compound 13

Method Info : ACE C8, 50x3mm, 3μ, 10-97% MeCN, 3min; 1ml/min, A: 0.1% TFA, B:MeCN.

Sample Info : Walkup method: 'A1097-3'
Target:

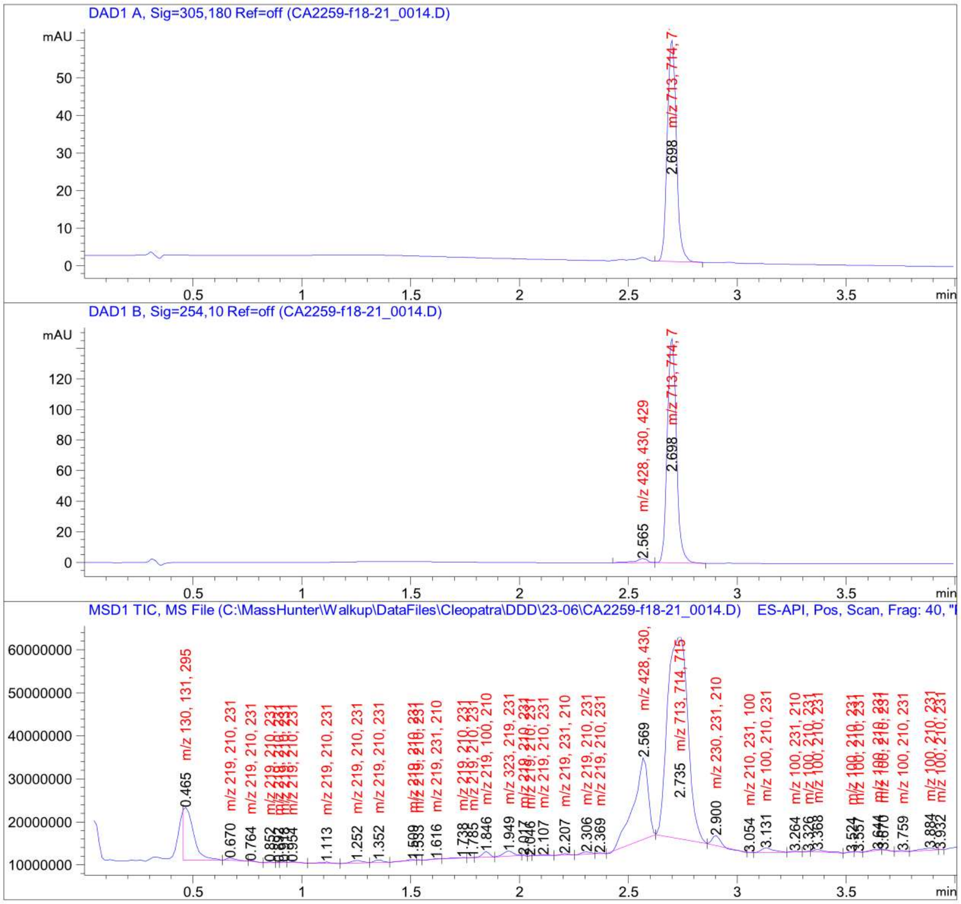

Signal 1: DAD1 A, Sig=305,180 Ref=off

Peak #	RetTime [min]	Type	Width [min]	Area [mAU*s]	Height [mAU]	Area %
1	2.698	BB	0.0460	172.45583	59.21549	100.0000

Totals : 172.45583 59.21549

Signal 2: DAD1 B, Sig=254,10 Ref=off

Peak #	RetTime [min]	Type	Width [min]	Area [mAU*s]	Height [mAU]	Area %
1	2.565	BB	0.0572	10.61201	2.74070	2.4158
2	2.698	BB	0.0460	428.65799	147.09473	97.5842

Totals : 439.27000 149.83543

HPLC traces of compound **14**

Compound 14

Method Info : ACE C8, 50x3mm, 3μ, 10-97% MeCN, 3min; 1ml/min, A: water 0.1% TFA B MeCN

Sample Info : Walkup method: 'A1097-3'
Target:

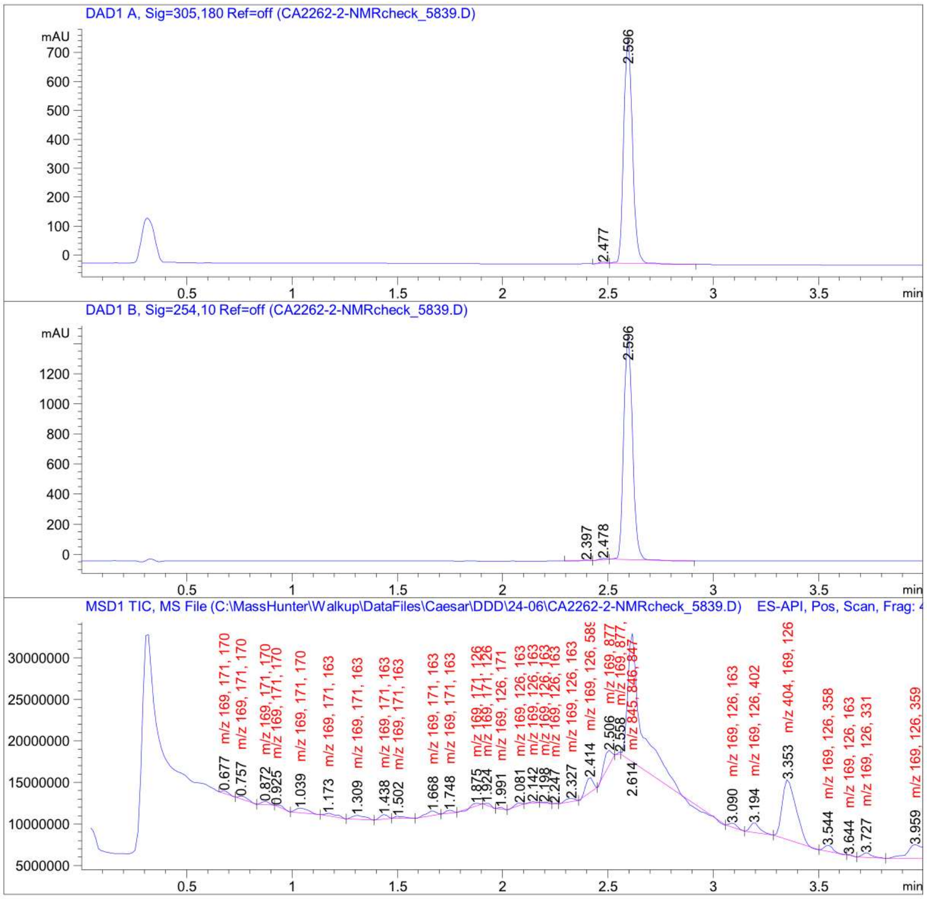

Signal 1: DAD1 A, Sig=305,180 Ref=off

Peak #	RetTime [min]	Type	Width [min]	Area [mAU*s]	Height [mAU]	Area %
1	2.477	BB	0.0377	8.44955	3.84056	0.3753
2	2.596	BB	0.0478	2242.67407	774.09235	99.6247
Totals :				2251.12362	777.93291	

Signal 2: DAD1 B, Sig=254,10 Ref=off

Peak #	RetTime [min]	Type	Width [min]	Area [mAU*s]	Height [mAU]	Area %
1	2.397	BB	0.0325	6.45880	3.66790	0.1488
2	2.478	BB	0.0370	18.50480	8.62307	0.4262
3	2.596	BB	0.0458	4316.57422	1488.75183	99.4250
Totals :				4341.53782	1501.04280	

HPLC traces of compound **15**

Compound 15

Method Info : ACE C8, 50x3mm, 3µ, 10-97% MeCN, 3min; 1ml/min, A: 0.1% TFA, B:MeCN.

Sample Info : Walkup method: 'A1097-3'
Target:

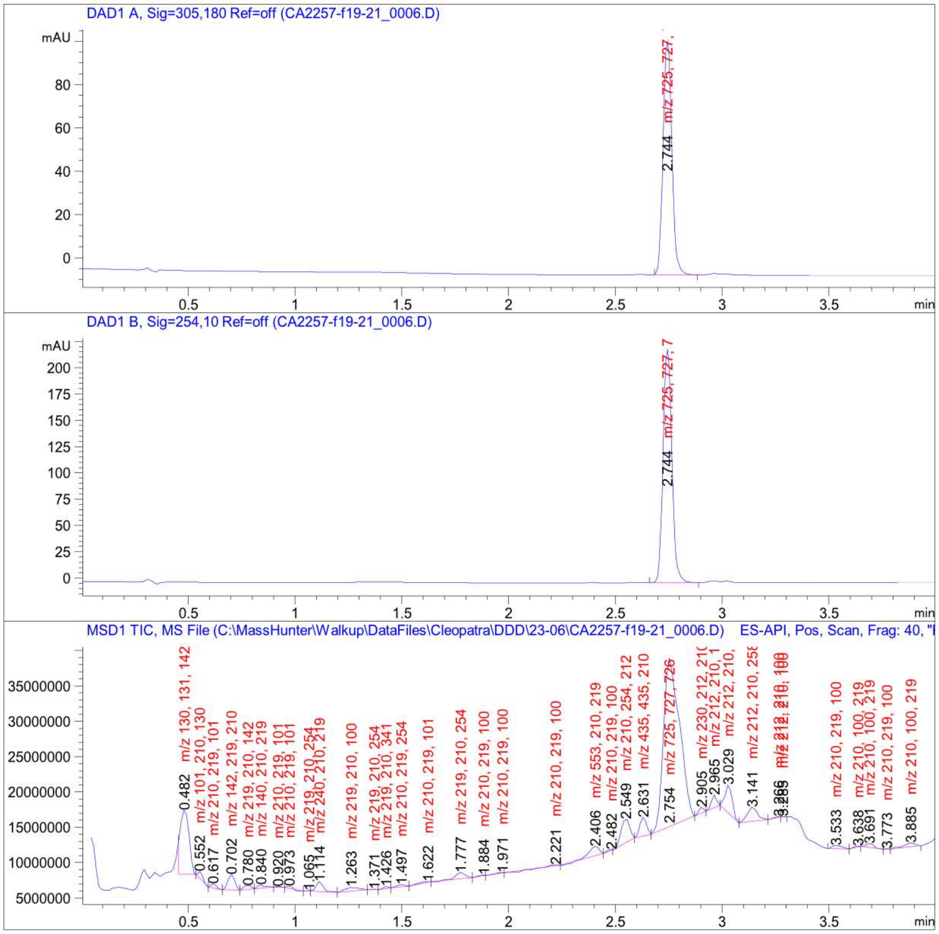

Signal 1: DAD1 A, Sig=305,180 Ref=off

Peak #	RetTime [min]	Type	Width [min]	Area [mAU*s]	Height [mAU]	Area %
1	2.744	BB	0.0476	311.44089	107.98170	100.0000
Totals :				311.44089	107.98170	

Signal 2: DAD1 B, Sig=254,10 Ref=off

Peak #	RetTime [min]	Type	Width [min]	Area [mAU*s]	Height [mAU]	Area %
1	2.744	BB	0.0477	638.63086	221.26765	100.0000
Totals :				638.63086	221.26765	

HPLC traces of compound **16**

Compound 16

Method Info : ACE C8, 50x3mm, 3µ, 10-97% MeCN, 3min; 1ml/min, A: water 0.1% TFA B MeCN

Sample Info : Walkup method: 'A1097-3'
Target:

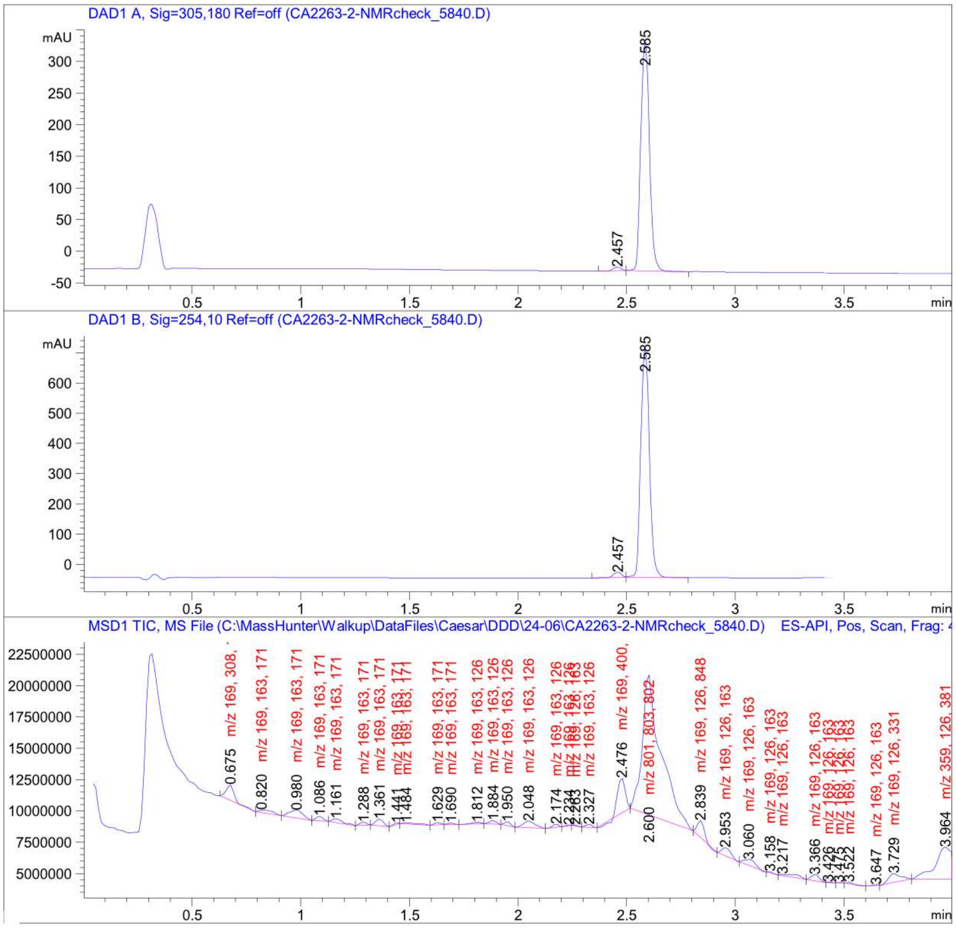

Signal 1: DAD1 A, Sig=305,180 Ref=off

Peak #	RetTime [min]	Type	Width [min]	Area [mAU*s]	Height [mAU]	Area %
1	2.457	BB	0.0421	13.73467	5.69816	1.3448
2	2.585	BB	0.0463	1007.59302	364.16425	98.6552

Totals : 1021.32769 369.86241

Signal 2: DAD1 B, Sig=254,10 Ref=off

Peak #	RetTime [min]	Type	Width [min]	Area [mAU*s]	Height [mAU]	Area %
1	2.457	BB	0.0393	38.94181	16.61147	1.8252
2	2.585	BB	0.0461	2094.66553	761.97919	98.1748

Totals : 2133.60734 778.59065

HPLC traces of compound **17**

Compound 17

Method Info : ACE C8, 50x3mm, 3µ, 10-97% MeCN, 3min; 1ml/min, A: water 0.1% TFA B MeCN

Sample Info : Walkup method: 'A1097-3'
Target:

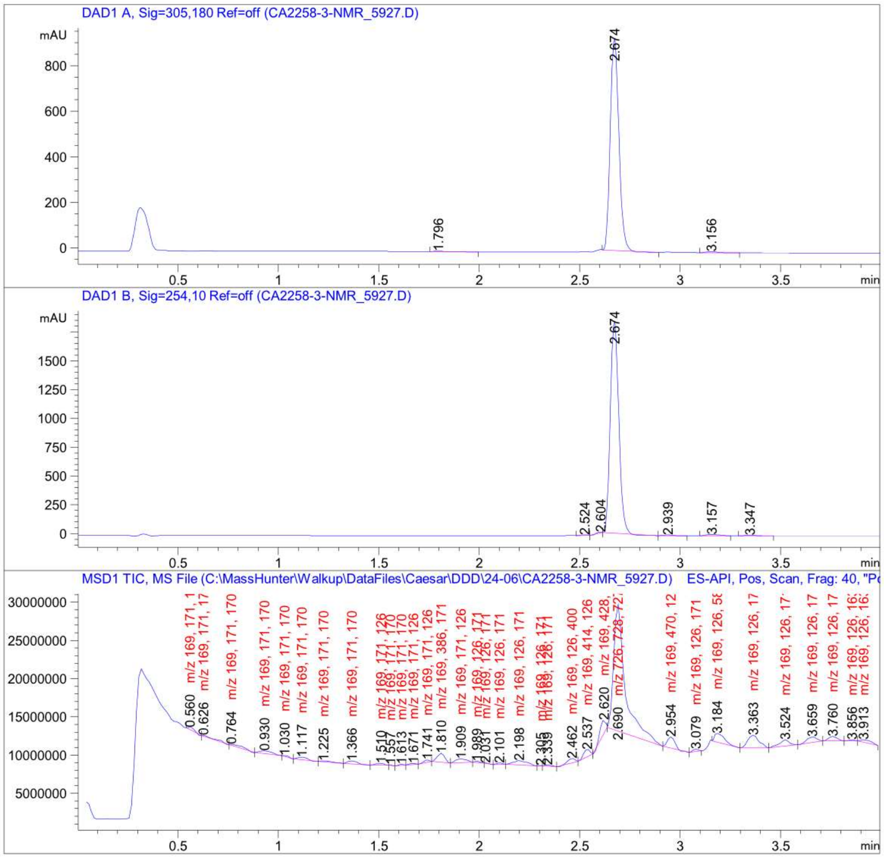

Signal 1: DAD1 A, Sig=305,180 Ref=off

Peak #	RetTime [min]	Type	Width [min]	Area [mAU*s]	Height [mAU]	Area %
1	1.796	BB	0.0371	5.70432	2.65439	0.2041
2	2.674	BB	0.0466	2771.73169	934.63287	99.1836
3	3.156	BB	0.0554	17.11089	4.81938	0.6123

Totals : 2794.54690 942.10664

Signal 2: DAD1 B, Sig=254,10 Ref=off

Peak #	RetTime [min]	Type	Width [min]	Area [mAU*s]	Height [mAU]	Area %
1	2.524	BB	0.0371	7.85541	3.64839	0.1441
2	2.604	BB	0.0234	8.33405	7.06892	0.1529
3	2.674	BB	0.0459	5374.12646	1848.30457	98.5894
4	2.939	BB	0.0438	12.46474	4.56845	0.2287
5	3.157	BB	0.0571	40.72761	11.02484	0.7472
6	3.347	BB	0.0542	7.50941	2.07582	0.1378

Totals : 5451.01769 1876.69099

Reaction schemes

Reaction schemes for compounds **11-17**

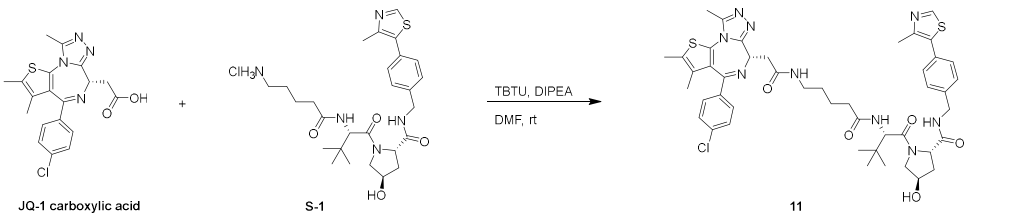

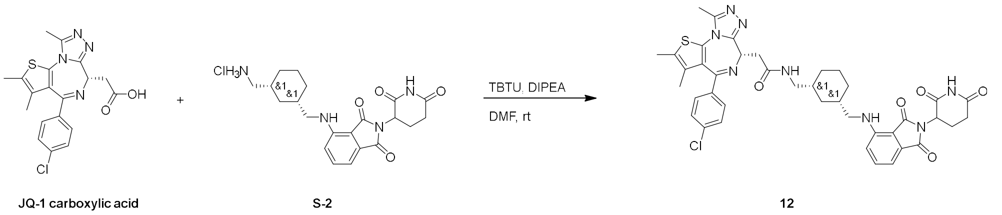

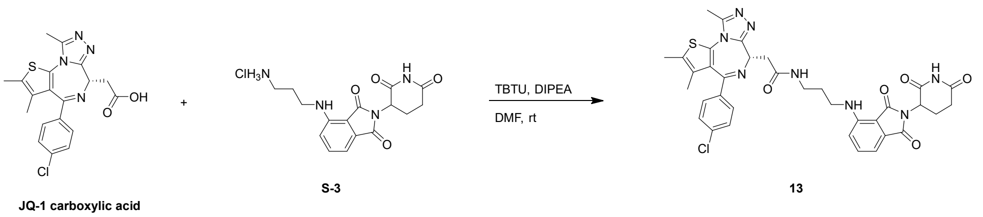

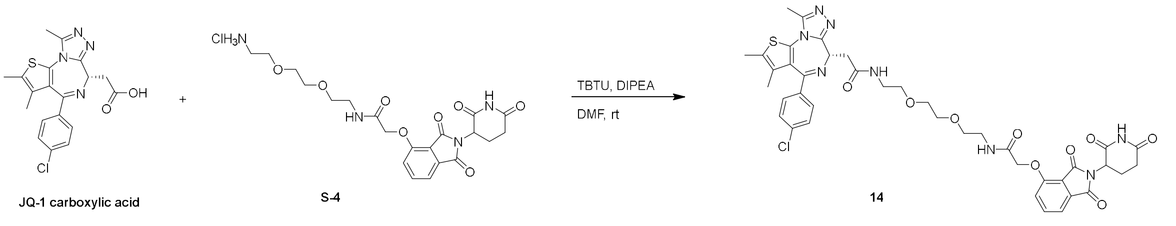

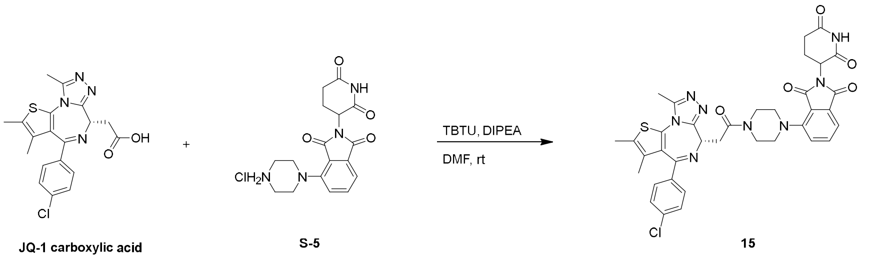

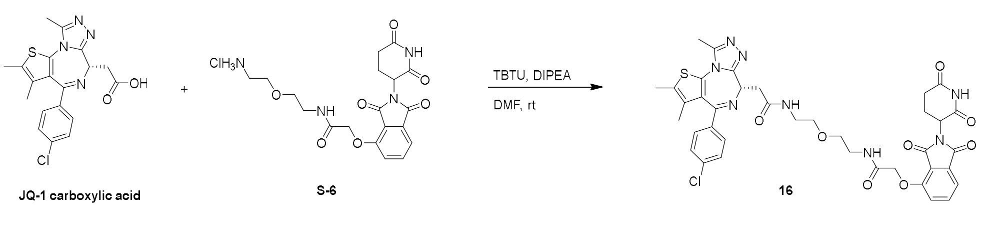

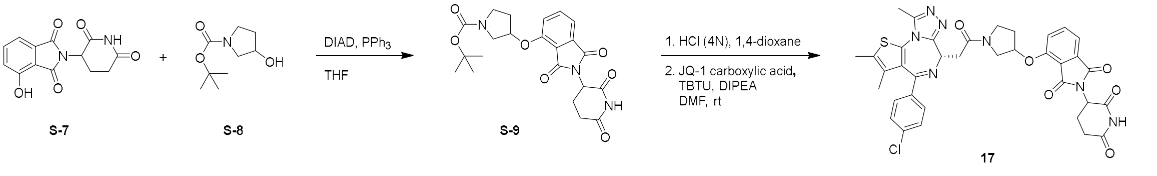

Supplement: Supplementary file 1 [file jm5c01499_si_001.pdf]
